# Biochar-mediated changes in the microbial communities of rhizosphere soil alter the architecture of maize roots

**DOI:** 10.3389/fmicb.2022.1023444

**Published:** 2022-10-04

**Authors:** Han Yan, Mengfei Cong, Yang Hu, Chunchen Qiu, Zailei Yang, Guangmu Tang, Wanli Xu, Xinping Zhu, Xia Sun, Hongtao Jia

**Affiliations:** ^1^Xinjiang Agricultural University, College of Resources and Environment, Urumqi, China; ^2^Xinjiang Key Laboratory of Soil and Plant Ecological Processes, Urumqi, China; ^3^Institute of Soil and Fertilizer and Agricultural Sparing Water, Xinjiang Academy of Agricultural Science, Urumqi, China; ^4^Key Laboratory of Saline-Alkali Soil Improvement and Utilization (Saline-Alkali Land in Arid and Semi-Arid Regions), Ministry of Agriculture and Rural Affairs, Urumqi, China

**Keywords:** biochar, microbial community, maize, root architecture, rhizosphere

## Abstract

Aeolian sandy soil is a key resource for supporting food production on a global scale; however, the growth of crops in Aeolian sandy soil is often impaired due to its poor physical properties and lack of nutrients and organic matter. Biochar can be used to enhance the properties of Aeolian sandy soil and create an environment more suitable for crop growth, but the long-term effects of biochar on Aeolian sandy soil and microbial communities need to be clarified. Here, a field experiment was conducted in which biochar was applied to a maize (*Zea mays* L.) field in a single application at different rates: CK, 0 Mg ha^−1^; C1, 15.75 Mg ha^−1^; C2, 31.50 Mg ha^−1^; C3, 63.00 Mg ha^−1^; and C4, 126.00 Mg ha^−1^. After 7 years of continuous maize cropping, verify the relationship between root architecture and soil microbial communities under biochar application using a root scanner and 16S/ITS rRNA gene sequencing. The application of biochar promoted the growth of maize. Specifically, total root length, total root surface area, total root volume, and root biomass were 13.99–17.85, 2.52–4.69, 23.61–44.41, and 50.61–77.80% higher in treatments in which biochar was applied (C2, C3, and C4 treatments) compared with the control treatment, respectively. Biochar application increased the diversity of bacterial communities, the ACE index, and Chao 1 index of C1, C2, C3, and C4 treatments increased by 5.83–8.96 and 5.52–8.53%, respectively, compared with the control treatment, and significantly changed the structure of the of bacterial communities in rhizosphere soil. However, there was no significant change in the fungal community. The growth of maize roots was more influenced by rhizosphere bacteria and less by fungal community. A microbial co-occurrence network revealed strong associations among rhizosphere microorganisms. The core taxa (Module hubs taxa) of the bulk soil microbial co-occurrence network were closely related to the total length and total surface area of maize roots, and the core taxa (Connectors taxa) of the rhizosphere soil were closely related to total root length. Overall, our findings indicate that the application of biochar promotes the growth of maize roots in aeolian sandy soil through its effects on bacterial communities in rhizosphere soil.

## Introduction

Aeolian sand soil is one of the important reserve resources of cultivated in the world (Ge et al., 2015). This soil type is mainly present in areas with low precipitation, large diurnal temperature fluctuations, and sandstorms, such as deserts, grasslands, and semi-desert grasslands (Driessen et al., 2001). Approximately 18% of China’s land area (1.74 × 10^6^ hm^2^) has aeolian sandy soil, and the area with aeolian sandy soil continues to grow (Kari et al., 2021). However, there are major challenges to growing crops in aeolian sandy soil because of its low content of organic matter and nutrients, as well as its poor water and fertilizer retention properties (Han et al., 2021). There is thus a need for more studies to explore the efficacy of using different approaches to enhance the properties of aeolian sandy soil.

Biochar is one potentially effective approach for enhancing the properties of aeolian sandy soil. Biochar is a solid, carbon (C)-rich product that is highly stable in soil, and it is produced *via* the high-temperature pyrolysis of biomass materials, including crop straw, rice husk, and livestock manure, under anoxic conditions (Sohi et al., 2010). The amount of straw produced on a global scale is substantial; straw is rich in nutrients, as it contains nearly half of the nutrients absorbed by crops (Lal, 2005). However, straw is often discarded and burned rather than used in crop production, and this practice results in an unnecessary waste of resources, as well as environmental pollution (Langmann et al., 2009). The reuse of straw to make biochar can reduce the environmental pollution associated with straw burning and enhance the properties of soil when biochar is applied to the soil (Zhang et al., 2021). Biochar has a loose and porous structure, and the physical properties of soil change following its application to soil (Soinne et al., 2014). For example, biochar can enhance the aeration and water-holding properties of soil (Glaser et al., 2002), increase the specific surface area and porosity of soil, and reduce soil bulk density (Novak et al., 2009; Busschei et al., 2010). Biochar is also rich in C and nutrients; thus, the application of biochar to soil can substantially increase the C content of soil and promote the conversion of soil C, nitrogen (N), and phosphorus (P; Lehmann et al., 2006). Biochar can also make the soil environment more suitable for the growth of soil microorganisms, promote the metabolic activities of soil microbes (Zhu et al., 2017), and increase the abundance and diversity of microbial communities (Siedt et al., 2021).

Several studies have shown that biochar application can have a substantial effect on soil microbial communities (Anderson et al., 2011; Luo et al., 2017; Hu et al., 2020; Akhil et al., 2021). Soil microbial populations were significantly increased in the long-term effect of biochar (Wardle et al., 2008; Kolb et al., 2009), but high application rates of biochar reduced soil microbial populations (Dempster et al., 2011). However, few studies have characterized the effects of biochar application on the microbial communities in rhizosphere soil (i.e., the root–soil interface). Biochar application can increase the biomass of pine roots and maize roots by 300% (Wardle et al., 1998) and from 88 to 92% (Yamato et al., 2006), respectively. Biochar can also have direct and indirect effects on the structure and diversity of soil microbial communities in the rhizosphere (Kolton et al., 2011; Yu et al., 2018). For example, biochar application was shown to increase the relative abundance of *Pseudomonas*, *Bacillus*, and *Trichoderma* in rhizosphere soil in a 6-week pot experiment (Jaiswal et al., 2018a). Biochar application was also shown to lead to significant increases in the diversity and evenness of rhizosphere bacterial communities in a 3-month experiment (Graber et al., 2010). The application of biochar over 4 consecutive years had a substantial effect on the structure of the soil fungal community; however, biochar application had no noticeable effect on fungal diversity (Yin et al., 2021). Overall, biochar application changes the soil physical (Lu et al., 2014; Nelissen et al., 2015) and chemical properties (Kimetu and Lehmann, 2010). The improvement of soil nutrient content can directly promote plant root growth (Abiven et al., 2015). In addition, biochar application can also change the soil microbial community by altering soil properties (Ding et al., 2016). In turn, soil microorganisms can act on crop roots (Bourceret et al., 2022). However, few studies have examined the long-term effects of biochar, determined the most appropriate application rate of biochar, as well as the relationship between root architecture and soil microbial communities under biochar application.

Here, we aimed to (1) identify the most suitable biochar application rate for fertilizing aeolian sandy soil; (2) characterize the long-term effects of biochar addition on the properties of aeolian sandy soil, the architecture of crop roots, and the diversity and structure of microbial communities in bulk and rhizosphere soil; and (3) clarify the relationships among soil, crop root architecture, and microbial communities under biochar addition. To address these aims, we conducted a field experiment in which biochar was applied to a maize field with aeolian sandy soil. We then characterized changes in the properties of aeolian sandy soil, the architecture of maize roots, and microbial communities following 7 years of continuous cropping and a single biochar application.

## Materials and methods

### Overview of the study area

**Figure 1:**
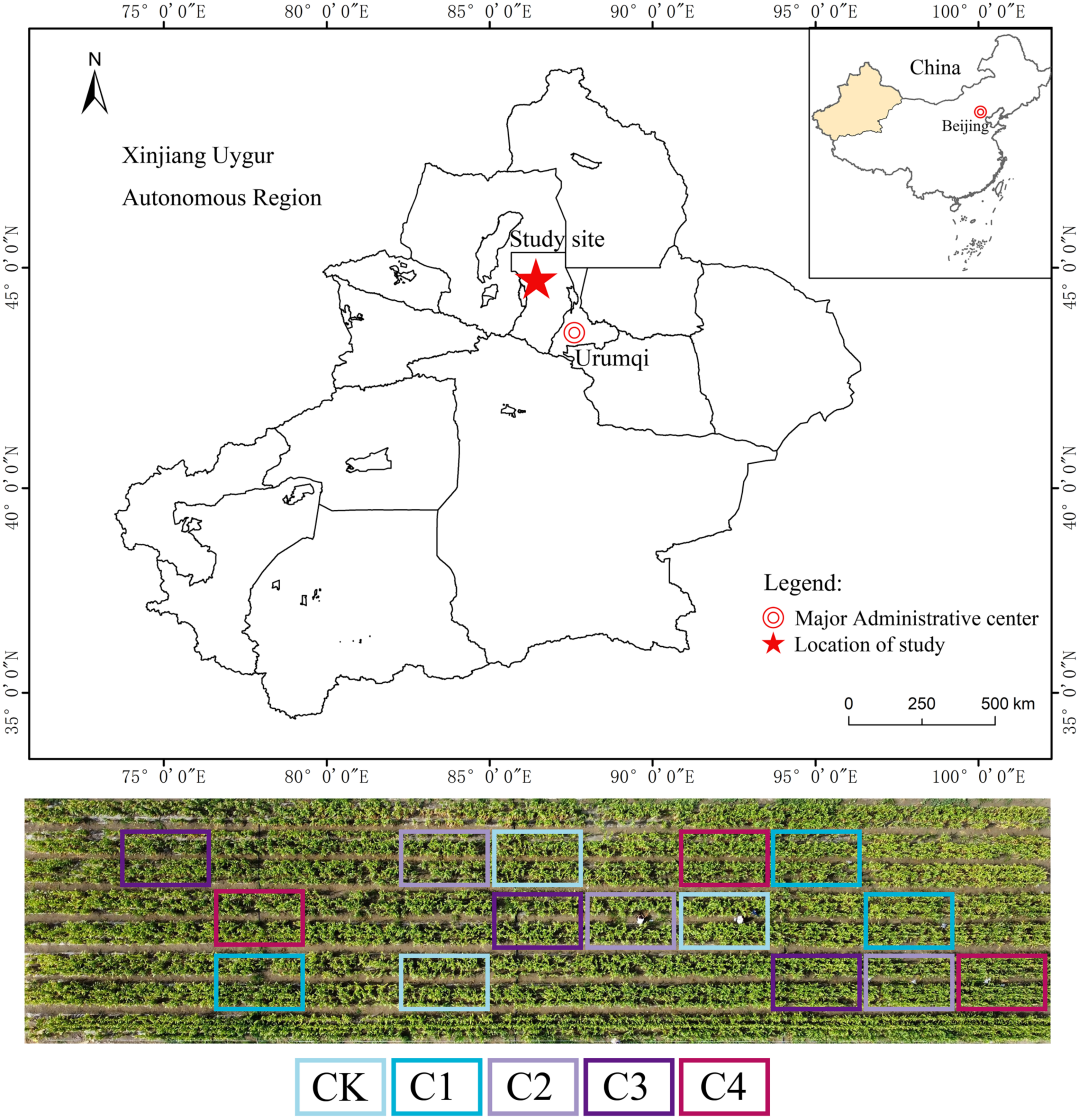
Study area within China.

Our study was conducted at the Battery Soil Improvement Experimental Station, 121 Regiment, Agricultural 8th Division, Shihezi Reclamation Area, Xinjiang Uygur Autonomous Region, China (43°26′–45°20′N, 84°58′–86°24′E) (Figure 1). The study area features an arid semi-desert climate with an average annual temperature of 7.5°C, 2,525 h of annual sunshine, a frost-free period of 169 days, 225 mm of annual rainfall, and 1,250 mm of annual evaporation. The aeolian sandy soil comprised 53.2% sand, 27.2% powder, and 19.6% clay grains (Ma, 2021).

### Experimental design

Our experiment was conducted on a mobile dune that was bulldozed in 2014. The experiment was conducted in a randomized group design with five treatments varying in the rate of biochar application: CK, 0 Mg ha^−1^; C1, 15.75 Mg ha^−1^; C2, 31.50 Mg ha^−1^; C3, 63.00 Mg ha^−1^; and C4, 126.00 Mg ha^−1^. There were three plots (4.6 m × 7 m) per treatment (Figure 1). Biochar was applied to each plot in separate applications, and it was mixed well with the soil at a depth of 0–30 cm (only applied only one time in 2014 layout experiment). Wheat straw was the source of the biochar used in the experiment. The biochar was carbonized at 450°C for 5 h, crushed, and filtered through a 2-mm mesh sieve. The properties of the biochar were as follows: pH, 8.21; organic C (OC), 1.38 g/kg; available nitrogen (AN), 7.40 mg/kg; available phosphorus (AP), 4.60 mg/kg; and available potassium (AK), 97.00 mg/kg. Maize (Xin Yu 53) was sown in May and harvested in September each year from 2014 and 2021. Only one crop was planted in our experimental plots per year; plants were irrigated *via* under-membrane drip irrigation; and fertilizer application and other management practices were based on those used by local farmers. The total amount of irrigation per year was 4800.0 m^3^ ha^−1^, and the total amount of fertilizer applied was 258 kg ha^−1^ N, 123 kg ha^−1^ P_2_O_5_, and 81 kg ha^−1^ K_2_O.

### Sample collection and processing

Many scholars found increased microbial diversity and a more uniform distribution of bacterial communities at the rhizosphere level during maturation (Schmidt and Eickhorst, 2014; Kwak et al., 2018; Schmidt et al., 2018). Therefore, we chose to collect soil samples from maize stage R6 (black layer) in 2021. Firstly, maize roots were collected. To avoid interactions between plots, two maize plants were randomly selected in the center of each plot (3.6 m × 5.0 m; ensure that the sampling interval of each plot is two meters apart), and then rectangular soil blocks (30 cm × 30 cm × 30 cm) were cut vertically downward around the maize roots. After slapping away large chunks of soil, and carefully separate the roots from the soil (Nazih et al., 2001). The collected maize roots were brought back to the laboratory. Secondly, when collecting maize roots, rhizosphere soil is collected by shaking it off from the roots in the air (Wang et al., 2009). One of these samples was placed in a self-sealing bag, all other plant residues in the soil samples were removed, air dried, and sieved (1 and 0.25 mm) for subsequent determination of basic rhizosphere soil chemical properties. The other sample was immediately placed in a sterile centrifuge tube (2 ml) and stored in a liquid nitrogen tank at −80°C for subsequent characterization of the soil microbial community. There were six replicates for each treatment.

Thirdly, bulk soil samples were collected. Also due to avoid interactions between plots, we selected the center of each plot (3.6 m × 5.0 m) as the collection area and collected two mixed soil samples using the five-point sampling method at a depth of 0–30 cm. One of the samples was placed in self-sealing bag, all other plant residues in the soil samples were removed, air dried, and sieved (1 and 0.25 mm) for subsequent determination of basic bulk soil chemical properties. Another sample was placed in sterile centrifuge tube (2 ml) in liquid N tanks at −80°C for subsequent characterization of soil microbial communities. Finally, we also collected two ring knife samples from each plot to determine the physical properties of the soil. Six replicate samples were taken for each treatment.

### Basic physicochemical properties of soil and maize root architecture

The collected plant roots were brought back to the laboratory, and the soil particles on the root surface were carefully washed with water. Images of maize roots were digitized using an Epson Perfection V850 Pro scanner, and WinRHIZO software was used to measure total root length, total root surface area, and total root volume. Root biomass is the weight recorded after drying at 105°C until reaching constant weight. A pH meter (Mettler Toledo FE28-Standard, Switzerland) was used to measure soil pH at water: soil ratio of 2.5:1. The H_2_SO_4_–K_2_Cr_2_O_7_ external heating method (Hu et al., 2022), alkali diffusion method, spectrophotometry (Shimadzu UV-1780, Japan; Shao et al., 2019), flame photometry (Shanghaiyuefeng FP6400, China; Chen et al., 2021), the drying method, H_2_SO_4_-HClO_4_ digestion—spectrophotometry (Shimadzu UV-1780, Japan), H_2_SO_4_-HClO_4_ digestion—flame photometry (Shanghaiyuefeng FP6400, China; Mei et al., 2021), and an elemental analyzer (Euro EA3000, Italy) were used to measure the content of SOC, AN, AP, AK, soil moisture content, total P (TP), total K (TK), and total N (TN), respectively.

### DNA extraction and sequencing

Total DNA was extracted using the Power Soil DNA Isolation Kit Power DNA Extraction Kit, and DNA integrity and purity were examined. The V3-V4 region of the bacterial 16S rRNA gene was amplified using the primers 338F (5′-ACTCCTAGGGAGGAGCA-3′) and 806R (5′-GGACTCHVGGGTWTTAT-3′) and combined with adapter sequences and barcode sequences (Quast et al., 2013). The fungal ITS1 gene was amplified using the primers ITS1 (5′-CTGTCATTAGGGAGAGAGA-3′) and ITS2 (5′-GCTGCGTTCTTCA TCGATGA-3′) and combined with adapter and barcode sequences (Kõljalg et al., 2013). PCR reactions were conducted in 50-μl systems with 100 ng of template DNA, 1.5 μl of primer (10 μmol/L), 25 μl of 2× PCR buffer for KOD FX Neo (Toyobo, Japan), 1.0 μl of KOD FX Neo DNA polymerase (1.0 U/μl; Toyobo, Japan), and 10 μl of dNTP (2 mmol/L). The thermal cycling conditions were as follows: pre-denaturation at 95°C for 5 min; 25 cycles of denaturation at 95°C for 40 s, annealing at 55°C for 40 s, and extension at 72°C for 40 s; and a final extension at 72°C for 7 min. A 1.8% agarose gel electrophoresis was used to detect the PCR products; an Illumina HiSeq 2500 platform was then used to sequence the quality-checked libraries.

### Bioinformatics analysis

The reads for each sample were spliced into tags according to the overlap among reads using FLASH software (version 1.2.11, http://ccb.jhu.edu/software/FLASH/); these raw tags were then filtered using Trimmomatic software (version 0.33) to obtain high-quality tags. The final data were obtained after chimeric sequences were removed using UCHIME (version 8.1). USEARCH software (version 10.0) was used to cluster the tags at the 97% similarity level. Operational taxonomic units (OTUs) were annotated using the Silva taxonomic database (Release 132, http://www.arb-silva.de) for bacterial OTUs and the Unite taxonomic database (Release 8.0, https://unite.ut.ee/) for fungal OTUs. Taxonomic ranks were assigned using the RDP Classifier (version 2.2, http://sourceforge.net/projects/rdpclassifier/) with a minimum confidence estimate of 80%. Mothur (version v.1.30, http://www.mothur.org/) was used to analyze the diversity of microbial communities. Linear discriminant analysis (LDA) effect size (LefSe) analysis^1^ was used to determine the effect of biochar addition on the abundance of each component of the microbial communities. The logarithmic LDA score indicating significant differences was 3.0 (Zhou et al., 2019).

### Data analysis

R (version 4.0.2) was used to analyze the data, and the significance of differences among treatments (*p* < 0.05) was determined using a least significant difference test in the Agricolae package. The abundances of microbial communities were added to the histograms using Origin software. Microbial taxa with abundance greater than 0.1% were selected, and microbial co-occurrence networks and Zi (intra-network module connectivity)-Pi (inter-network module connectivity) plots were made using the igraph package in R. Here, Network hubs (Zi > 2.5; Pi > 0.62), Module hubs (Zi > 2.5; Pi ≤ 0.62), connectors (Zi ≤ 2.5; Pi > 0.62), and peripherals (Zi ≤ 2.5; Pi ≤ 0.62) were defined according to their Zi and Pi threshold value (Poudel et al., 2016). Network hubs, Module hubs, and connectors mean the nodes were highly connected within or between modules, and he can act as a core taxa (Guimerà and Amaral, 2005; Toju et al., 2018). Correlations of soil microbial communities with soil physicochemical properties and maize root architecture were determined using the corrplot software package.

## Results

### Soil properties and maize roots affected by biochar application

The application of biochar had a significant effect on the soil moisture content and soil bulk density (Supplementary Table S1). The soil moisture content was 3.09% lower in the C4 treatment than in the CK treatment. Soil bulk density was 0.34, 1.45, 2.04, and 2.34% lower in the C1, C2, C3, and C4 treatments than in the CK treatment, respectively.

In the bulk soil, biochar application altered the content of AN, AP, total phosphorus (TP), AK, and total potassium (TK; Supplementary Table S2). The soil AN content was 36.72, 65.91, 138.79, and 143.88% higher in the C1, C2, C3, and C4 treatments than in the CK treatment, respectively, and these differences were significant. The content of AP was 12.60 and 42.00% higher in the C3 and C4 treatments than in the CK treatment, respectively, and these differences were significant. The content of TP was 7.14 and 14.29% higher in the C3 and C4 treatments than in the CK treatment, respectively, and these differences were significant. The AK content was 15.84, 17.83, and 28.96% higher in the C2, C3, and C4 treatments than in the CK treatment, respectively. The TK content was 20.88% higher in the C4 treatment than in the CK treatment, and this difference was significant.

In the rhizosphere soil, the SOC content was 17.00 and 23.85% higher in the C3 and C4 treatments than in the CK treatment, respectively, and these differences were significant; however, there were no significant differences in the pH and TN among treatments (Supplementary Table S2). The AN, AP, AK, TP, and TK content were higher in the C1, C2, C3, and C4 treatments than in the CK treatment, and these differences were significant. Specifically, AN was 10.53, 46.22, 55.92, and 106.58% higher; AP was 8.10, 19.25, 27.34, and 35.94% higher, and AK was 13.14, 18.58, 18.24, and 53.31% higher in the C1, C2, C3, and C4 treatments than in the CK treatment, respectively.

**Figure 2:**
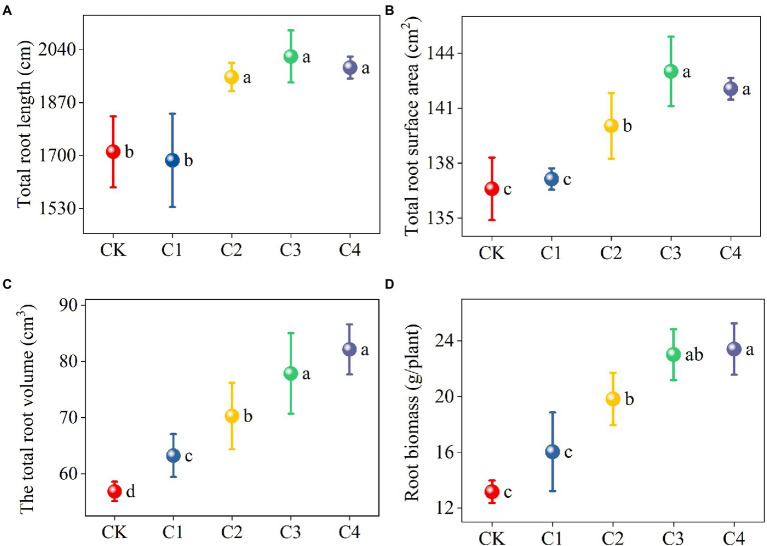
Effect of biochar addition on the architecture of maize roots. Different lowercase letters indicate significant differences under different levels of biochar application (*p* < 0.05). **(A)** Effect of biochar addition on the total root length of maize. **(B)** Effect of biochar addition on the total root surface area of maize. **(C)** Effect of biochar addition on the total root volume of maize. **(D)** Effect of biochar addition on the root biomass of maize.

The application of biochar had a significant effect on the architecture of maize roots (Figure 2). The total root length and total root surface area of maize were significantly higher in the C2, C3, and C4 treatments than in the CK treatment. Specifically, the total root length was 13.99, 17.85, and 15.78% higher and the total root surface area was 2.52, 4.69, and 3.99% higher in the C2, C3, and C4 treatments than in the CK treatment, respectively. The total root volume was 11.21, 23.61, 36.92, and 44.41% higher in the C1, C2, C3, and C4 treatments than in the CK treatment, respectively, the root biomass was 50.61, 74.78, and 77.80% higher in the C2, C3, and C4 treatments than in the CK treatment, respectively, and these differences were significant.

### Microbial community diversity affected by biochar application

**Figure 3:**
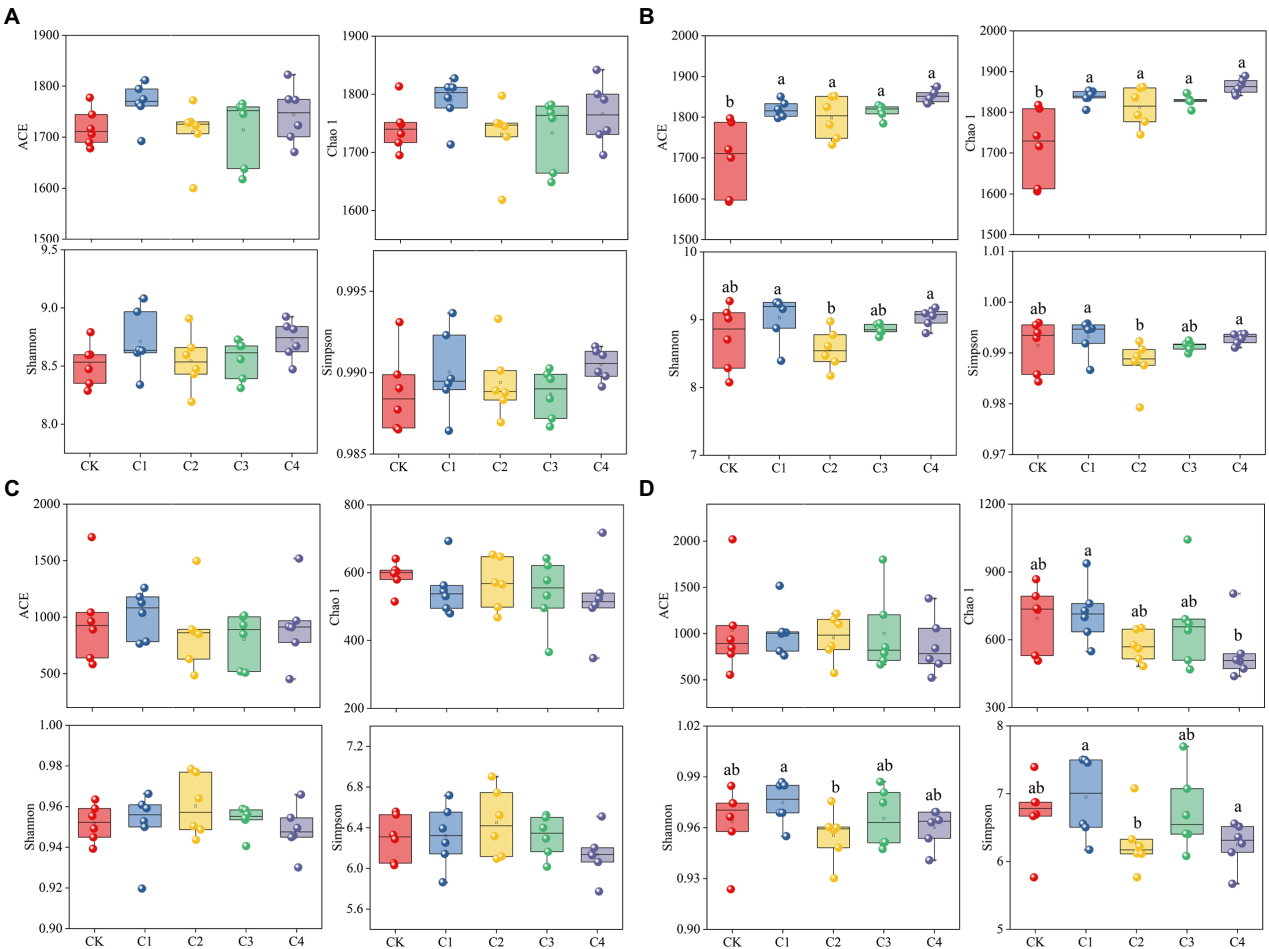
Effect of biochar addition on the α-diversity of soil microbial communities. **(A)** α-diversity of the bacterial communities in bulk soil. **(B)** α-diversity of the bacterial communities in rhizosphere soil. **(C)** α-diversity of the fungal communities in bulk soil. **(D)** α-diversity of the fungal communities in rhizosphere soil. Different lowercase letters indicate significant differences among biochar application treatments (*p* < 0.05).

The addition of biochar had no significant effect on the diversity of bacterial and fungal communities in bulk soil; however, biochar addition had a significant effect on the diversity of bacterial and fungal communities in rhizosphere soil (Figure 3). The ACE index was 7.05, 5.83, 6.79, and 8.96% and the Chao 1 index was 6.98, 5.52, 6.44, and 8.53% higher in the C1, C2, C3, and C4 treatments than in the CK treatment for bacterial communities in rhizosphere soil, respectively (Figure 3B). No significant differences in the ACE, Chao 1, Simpson, and Shannon indexes of the fungal communities were observed among biochar treatments and the CK treatment (Figure 3D). However, the Chao 1 index was 24.14% lower in the C4 treatment than in the C1 treatment; the Simpson index was 1.99% lower in the C2 treatment than in the C1 treatment; and the Shannon index was 9.75 and 10.04% lower in the C2 and C4 treatments than in the C1 treatment, respectively.

### Microbial community structure affected by biochar application

**Figure 4:**
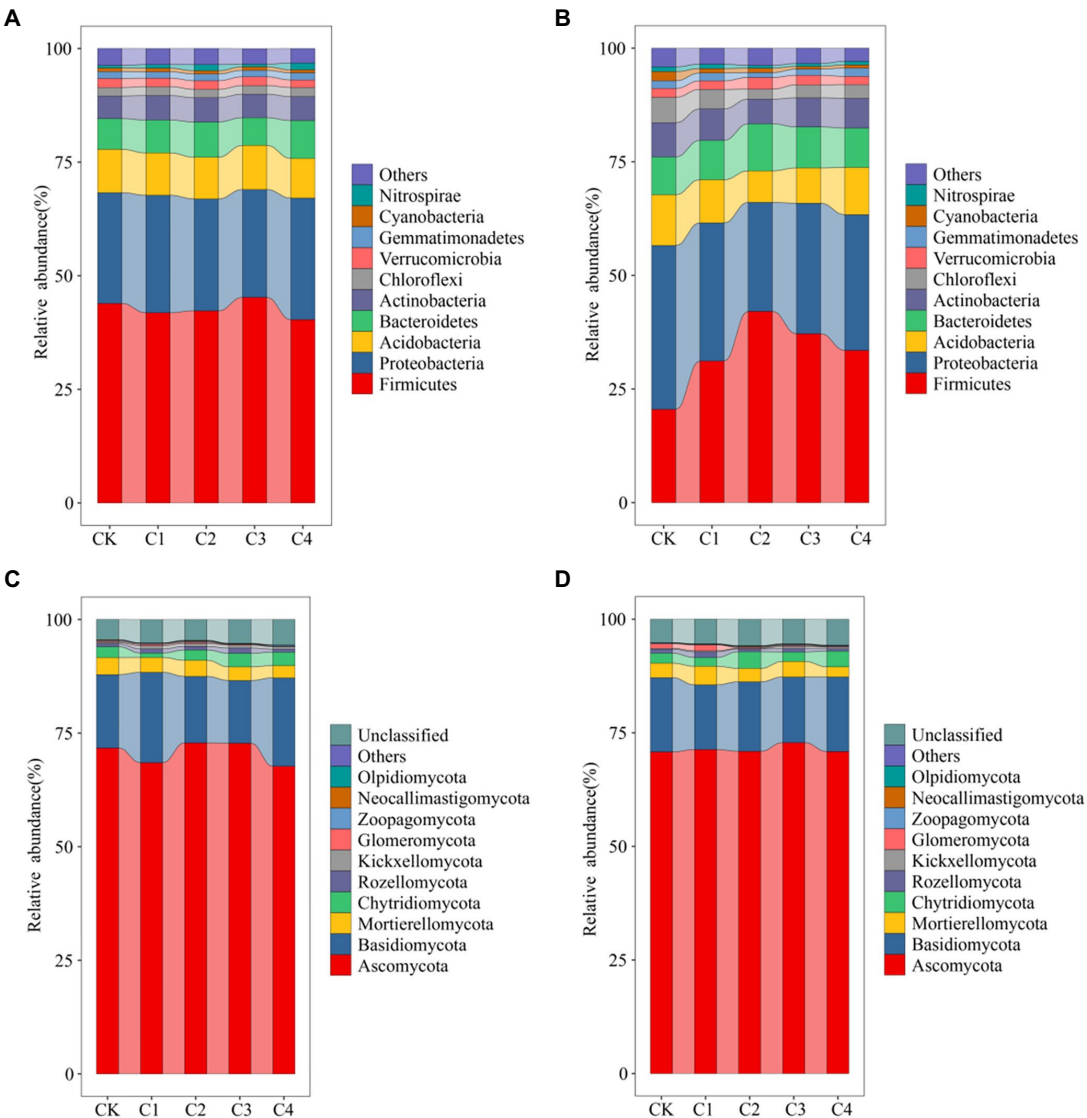
Effect of biochar addition on the structure of soil bacterial communities. **(A)** Bacterial communities in bulk soil at the phylum level (TPO10). **(B)** Bacterial communities in rhizosphere soil at the phylum level (TPO10). **(C)** Fungal communities in bulk soil at the phylum level (TPO10). **(D)** Fungal communities in rhizosphere soil at the phylum level (TPO10).

Changes in the top 10 bacterial phyla in terms of relative abundance (Figure 4) were characterized in the soil samples from the different treatments. Variation in the structure of the bacterial community was low in bulk soil (Figure 4A). Firmicutes was the most abundant phylum (average abundance of 42.73%), followed by Proteobacteria (average abundance of 25.07%). The structure of the bacterial community was more variable in rhizosphere soil (Figure 4B). The relative abundance of Firmicutes was 20.53, 31.16, 42.09, 37.17, and 33.51% in the CK, C1, C2, C3, and C4 treatments, respectively. The relative abundance of Proteobacteria was 36.06, 30.41, 23.98, 28.71, and 29.86% in the CK, C1, C2, C3, and C4 treatments, respectively. The structure of fungal communities was less variable in bulk and rhizosphere soil (Figures 4C,D). Ascomycota was the most abundant phylum (average abundance of 70.70%), followed by Basidiomycota (average abundance of 16.78%).

In this study, the top 50 genus in terms of relative abundance were selected to demonstrate the changes in bacterial and fungal community composition in bulk and rhizosphere soil (Supplementary Figures S1, S2). In bulk soil, *Lactobacillus* was the most abundant bacterial genus (average abundance of 15.09%). C4 treatment significantly altered bulk soil bacterial genus, such as *Akkermansia*, *Mycoplasma*, *Ensifer*, etc. (Supplementary Figure S1A). The structure of the bacterial community was more variable in rhizosphere soil. After biochar application, *Lactobacillus* abundance was significantly increased by 3.66, 7.75, 6.72, and 5.21% in C1, C2, C3, and C4 treatments, respectively. *Sphingomonas* abundance was significantly reduced by 1.81, 4.73, 1.91, and 1.63% in C1, C2, C3, and C4 treatments, respectively (Supplementary Figure S1B). An unclassified genus was the most abundant fungal genus (average abundance of 37.60%) in bulk soil (Supplementary Figure S2A). In rhizosphere soil, the average abundance of this genus was 32.55% (Supplementary Figure S2B).

**Figure 5:**
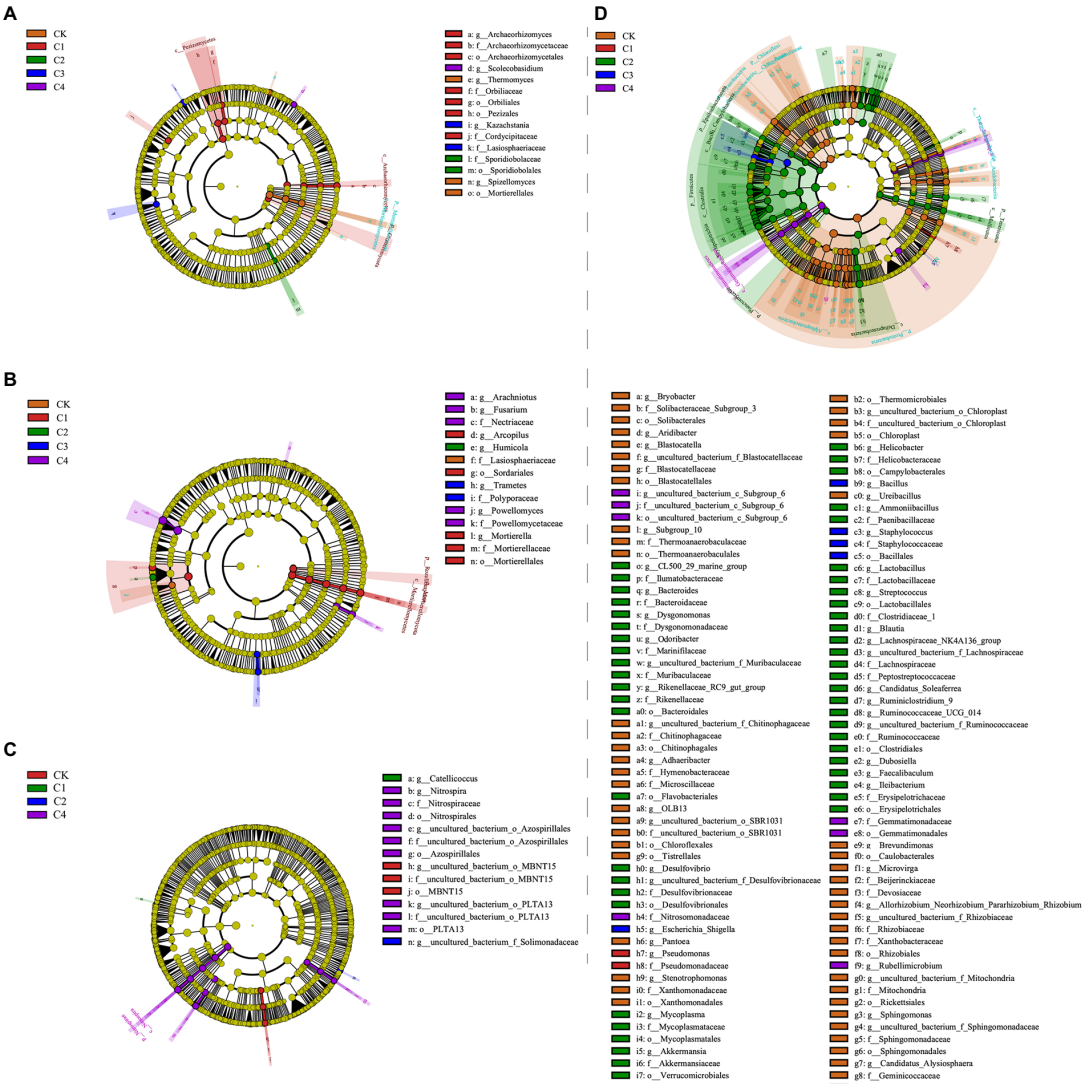
Analysis of the effect of biochar addition on soil microbial communities at the phylum and genus level using Linear discriminant analysis (LDA) effect size (LEfSe). **(A)** Fungal communities in bulk soil. **(B)** Fungal communities in rhizosphere soil. **(C)** Bacterial communities in bulk soil. **(D)** Bacterial communities in rhizosphere soil.

Linear discriminant analysis (LDA) effect size analysis revealed significant differences among treatments in 14 bacterial taxa (Figure 5C; Supplementary Figure S1A) and 15 fungal taxa (Figure 5A; Supplementary Figure S2A) in bulk soil. In the rhizosphere soil, significant differences among treatments were observed in 14 fungal taxa (Figures 5B,D and Supplementary Figure S2B) and 114 bacterial taxa (Figure 5D and Supplementary Figure S1B). The largest difference observed between treatments was in Firmicutes, which was 21.56% more abundant in the C2 treatment than in the CK treatment.

### Correlation analysis of soil, microbial communities, and root architecture

**Figure 6:**
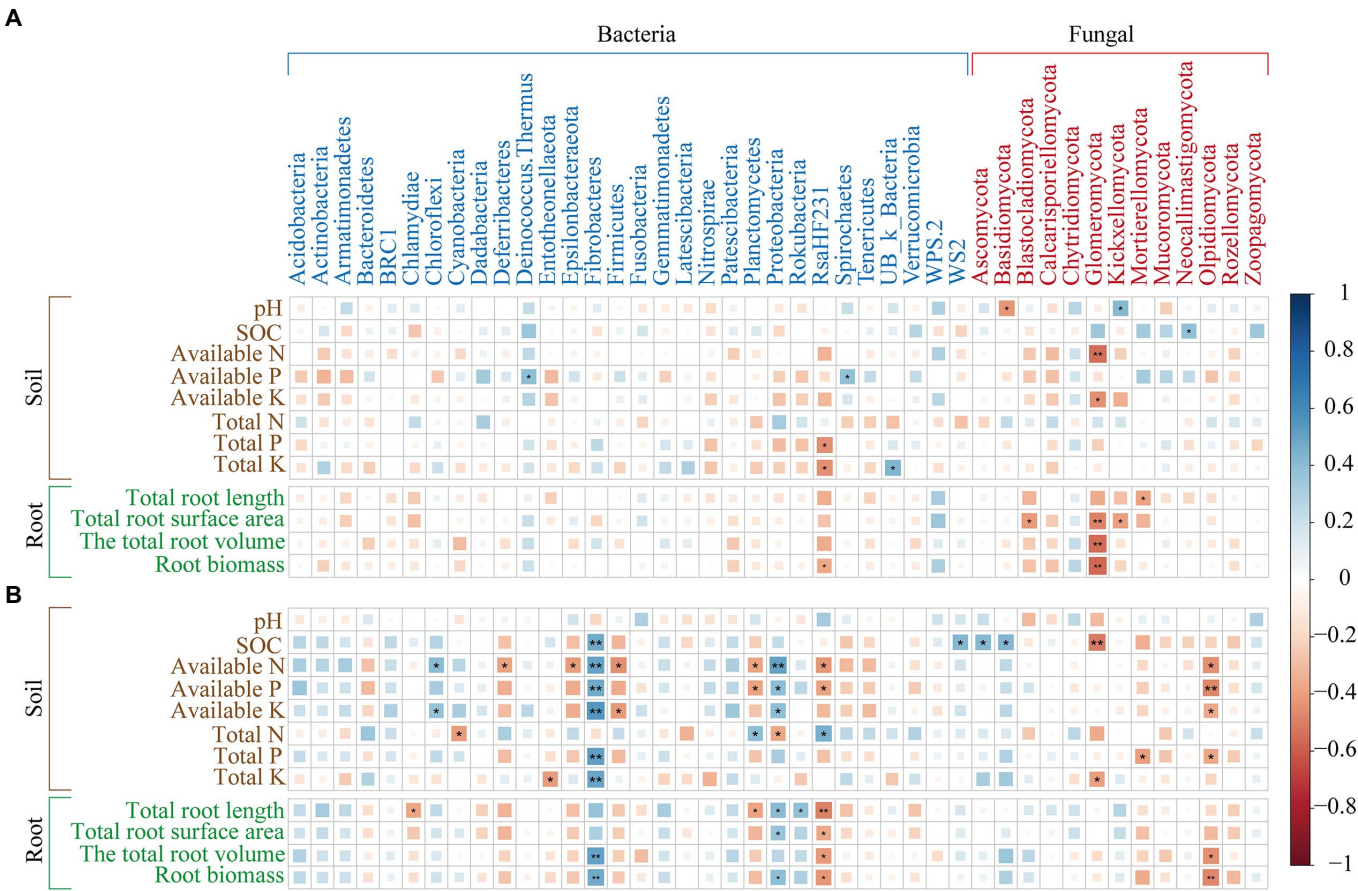
Relationships among soil microbial communities, soil physicochemical properties, and maize root architecture. **(A)** Communities in bulk soil. **(B)** Communities in rhizosphere soil. ^*^p < 0.05; ^**^p < 0.01.

Soil physicochemical properties and maize root architecture were highly correlated with microbial communities in rhizosphere soil at the phylum level; by contrast, correlations of soil physicochemical properties and maize root architecture with the microbial communities in bulk soil microbes at the phylum level were weak (Figure 6). In rhizosphere soil (Figure 6B), SOC, AN, AP, AK, TP, TK, total root volume, and root biomass were highly significantly and positively correlated with Fibrobacteres and negatively correlated with Olpidiomycota; AN, AP, and total root length were significantly and negatively correlated with Planctomycetes; and AN, AP, total root length, total surface area, total volume, and root biomass were significantly and negatively correlated with RsaHF231.

**Figure 7:**
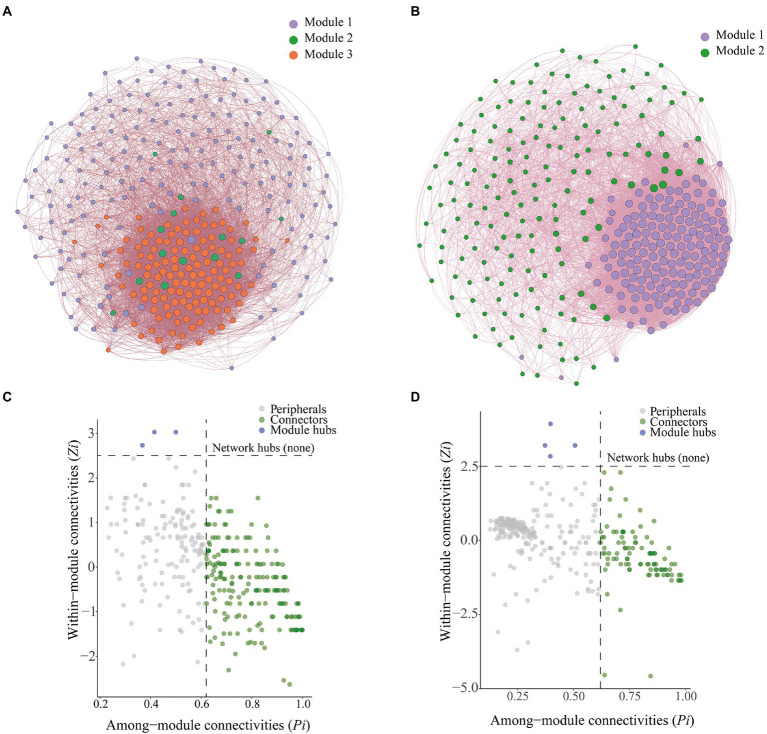
Co-occurrence networks and analysis of soil microbial communities. **(A)** Co-occurrence network of bacteria and fungi with relative abundance greater than 0.1% in bulk soil. **(B)** Co-occurrence network of bacteria and fungi with relative abundance greater than 0.1% in rhizosphere soil. **(C)** Zi-Pi plots of bacteria and fungi with relative abundance greater than 0.1% in bulk soil. **(D)** Zi-Pi plots of bacteria and fungi with relative abundance greater than 0.1% in rhizosphere soil.

We constructed a microbial co-occurrence network (Figure 7A) to clarify interrelationships among microorganisms. Bulk soil microorganisms could be divided into three modules, which were referred to as Module 1, Module 2, and Module 3. Rhizosphere soil microorganisms could be divided into two modules, which were referred to as Module 1 and Module 2 (Figure 7B). In the bulk soil group, the bacterial–fungal mutualistic network contained 328 nodes and 10,036 edges, and the average path length was 61.195; in the rhizosphere soil group, the bacterial–fungal mutualistic network contained 328 nodes and 14,472 edges, and the average path length was 88.244 (Table 1).

**Table 1:** Co-occurrence network analysis coefficients.

**Treatment**	**The number of nodes**	**The number of edges**	**Average degree**	**Weighted average**	**Mean clustering coefficient**	**Mean path length**
Bulk	328	10,036	61.195	87.762	0.466	2.023
Rhizosphere	328	14,472	88.244	155.682	0.568	1.912

Zi-Pi plots were constructed to identify the core OTUs in the microbial networks (Figures 7C,D). We identified a total of 189 connection points and three module centroids in bulk soil microbial network (Figure 7C). There were 98 connection points and four module centroids in the rhizosphere soil microbial network (Figure 7D). Ascomycota was the most abundant phylum (average abundance of 69.10%) among connector’s taxa in the bulk soil co-occurrence network, followed by Basidiomycota (average abundance of 12.98%). Ascomycota was the most abundant phylum among hubs taxa in the bulk soil co-occurrence network, and there was extensive variation in the abundance of Ascomycota among treatments; the relative abundance of Ascomycota was 62.64, 96.31, 70.92, 87.76, and 64.62% in the CK, C1, C2, C3, and C4 treatments, respectively. This was followed by Chytridiomycota, and its relative abundance was 37.36, 3.69, 29.08, 12.24, and 35.38% in the CK, C1, C2, C3, and C4 treatments, respectively. Ascomycota was the most abundant phylum (average abundance of 62.43%) among connectors taxa in the rhizosphere soil co-occurrence network, followed by Basidiomycota (average abundance of 9.53%). Basidiomycota was the most abundant phylum among hubs taxa in the rhizosphere soil co-occurrence network, and there was extensive variation in the abundance of Basidiomycota among treatments; the relative abundance of Basidiomycota was 65.93, 50.60, 57.58, 45.97, and 4.82% in the CK, C1, C2, C3, and C4 treatments, respectively. This was followed by Mortierellomycota, which had relative abundances of 34.07, 49.40, 42.42, 54.03, and 95.18% in the CK, C1, C2, C3, and C4 treatments, respectively.

**Figure 8:**
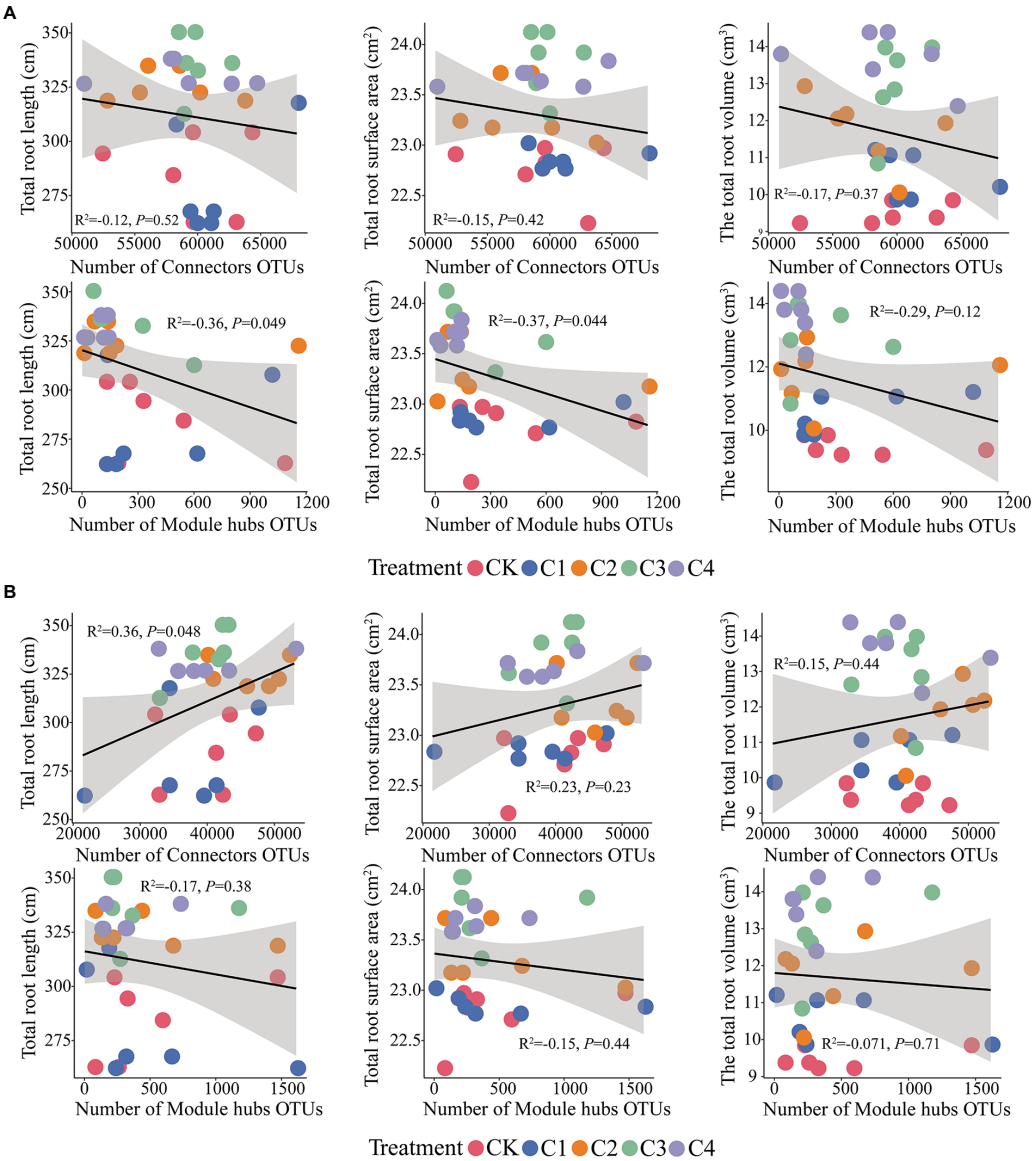
Effect of core microbial taxa on the architecture of maize roots. **(A)** Effect of microbial taxa in bulk soil on the architecture of maize roots. **(B)** Effect of microbial taxa in rhizosphere soil on the architecture of maize roots.

Connectors taxa were not significantly correlated (*p* > 0.05) with total root length, total root surface area, and total root volume (Figure 8); hubs taxa were significantly correlated (*p* < 0.05) with total root length and total root surface area in bulk soil (Figure 8A). In rhizosphere soil, connectors taxa were significantly correlated with total root length (*p* < 0.05); hubs taxa were not significantly correlated with total root length, total root surface area, and total root volume (*p* > 0.05; Figure 8B).

## Discussion

Soil microorganisms have a substantial effect on the flow of energy and material cycling of ecosystems. Studies of soil microbes have been a major focus in soil and environmental investigations (Yao et al., 2006; Todd-Brown et al., 2012). The plant–root environment has a major effect on microbial communities, and microbial communities can affect the growth of plant roots (Vejan et al., 2016; Trivedi et al., 2020). The application of biochar can make the soil environment more suitable for soil microorganisms and thus affect microbial activity (Li et al., 2020a). However, the relationship between crop root architecture and microbial communities under biochar addition has not been extensively studied. Here, we analyzed the long-term effects of biochar addition on the architecture of maize roots and microbial communities using 16S/ITS rRNA gene sequencing.

### Effect of biochar application on the architecture of maize roots

The results of our experiment showed that even a single application of biochar can have a significant effect on the growth of maize roots after 7 years. This is consistent with the results of several studies showing that the application of biochar enhances soil properties, which promotes the growth and development of roots (Abiven et al., 2015). However, the results of recent studies examining the effects of biochar application on the growth and development of plant roots are variable. In some studies, biochar has been shown to have a positive effect on the root growth of crops (Crane-Droesch et al., 2013). In other studies, biochar has been shown to have a negative effect on root growth, including toxic effects that force plants to grow more roots to meet their water and nutrient needs (Hodge, 2004; Peng et al., 2010). Our findings demonstrate that biochar has a positive effect on root growth (Figure 2), and the enhancement of the physicochemical properties of soil by biochar is one of the driving forces of this positive effect (Olmo et al., 2016). Correlation analysis between soil properties and root growth (Supplementary Figure S5) revealed a positive correlation between AP and TP in the rhizosphere soil and root growth. Previous studies have indicated that P reacts with various chemical and biological components in the soil and that increases in P alter maize root secretions and root symbionts, which in turn increases the growth of the lateral roots of maize (Lynch, 2011; Bourceret et al., 2022). This conclusion was confirmed by the changes in soil chemistry, where the application of biochar significantly increased the nutrient content, such as AP and TP in the rhizosphere soil (Supplementary Table S2), which provided the plant with nutrients required for root growth (Ding et al., 2016). However, soil bulk density was negatively correlated with root growth (Supplementary Figure S5). It showed that the total root length, total root surface area, and total root volume tended to increase as the soil bulk density decreased. This is in agreement with previous studies that a lower bulk density increases soil porosity leading to increased aeration, which affects root distribution and growth (Bengough and Young, 1993; Goodman and Ennos, 1999). Notably, we found that the application of biochar significantly reduced the soil moisture content of C4 treatment (Supplementary Table S1), which is inconsistent with previous studies, which found that the application of biochar improved the water retention and effective moisture of the soil (Baccile et al., 2009; Laird et al., 2010; Glab et al., 2018). Our study found that opposite results in administering doses of higher biochar (126.00 Mg ha^−1^). First of all, previous studies found that charcoal applications greater than 80.00 Mg ha^−1^ instead reduced soil water-holding properties, which may be due to the increase of soil aeration pore space and the decrease of capillary pore space, resulting in the decrease of soil water holding capacity (Gao et al., 2011; Carvalho et al., 2016). Secondly, maize is a deep-rooted crop with a root system that can grow up to 1 m deep. Excessive application of biochar in shallow Soils can affect the water holding capacity of the soil around the maize root system (Feng et al., 2021). Thirdly, the previous study was conducted after the fourth month and after the 39th day of biochar application, while our study was conducted after 7 years of biochar application (Laird et al., 2010; Glab et al., 2018) and aging biochar may affect soil moisture content. Finally, we also found that the biomass of maize roots was greatest when 126.00 Mg ha^−1^ biochar was applied (Figure 2), maize roots may have absorbed more water, resulting in the reduction of soil water content (Dardanelli et al., 2004).

Changes in the roots of plants might also be affected by the interaction between biochar and rhizosphere soil microbial communities (Glaser et al., 2002; Liang et al., 2006; Li et al., 2020a). The application of biochar increases the production of exudates by roots and provides nutrients and energy for microbial metabolism and growth, which alters the relationship between rhizosphere microorganisms and plants (Ma et al., 2019). These findings are consistent with the results of our study; rhizosphere soil microorganisms had a closer relationship with maize root growth than bulk soil microorganisms under biochar application (Figure 6). In addition, rhizosphere microorganisms accumulate around the root system and enhance the bioavailability of insoluble minerals, which increases the uptake of minerals by the roots and provides nutrients to the plant thus changing the maize root structure (Trivedi et al., 2020).

### Differences in rhizosphere soil and bulk soil bacterial and fungal communities

We found that biochar application had a more pronounced effect on rhizosphere soil bacterial communities than on fungal communities. Previous studies have shown that environmental factors have stronger effects on bacterial communities than on fungal communities (de Vries et al., 2018; Yang and Wu, 2020). This might stem from the fact that bacteria can be more readily adsorbed by biomass charcoal than fungi (Pietikäinen et al., 2000); bacteria can also more rapidly adapt to changes in soil nutrients associated with biomass charcoal compared with fungi (Lehmann et al., 2011). Several non-mutually exclusive explanations might explain these observations. Previous studies have suggested that biochar has an indirect effect on the growth of bacteria and a direct effect on the growth of fungi. Specifically, biochar species have direct effects on the abundance of fungi, whereas bacteria are primarily affected by changes in soil properties associated with biochar application (Yang and Wu, 2020). This is consistent with our finding that the application of biochar significantly enhanced soil physicochemical properties (Supplementary Tables S1, S2). The contents of SOC, AN, AP, and AK in rhizosphere soil increased after biochar application. Researchers have found similar results with Tobacco, biochar addition increased the richness and diversity of the bacterial community in the tobacco rhizosphere, which was related to the soil physical and chemical properties (Zhang et al., 2017). In addition, fungi degrade the recalcitrant C in biochar more readily than bacteria, can grow in the pores of biochar, and use additional resources (Lehmann et al., 2011). In this study, the length of time (7 years) since biochar application, the low content of recalcitrant C in biochar, and the destruction of the pores over time might explain the weak effect of biochar application on fungi in our study. Therefore, bacterial communities were more affected by biochar treatment than fungal communities.

We found that Firmicutes was the dominant bacterial phylum in the soils at our study site, and members of this phylum are known to be well adapted to survive extreme conditions (Hayward et al., 2010). The soils at our study site are sandy, poor in nutrients, and low in organic matter (Han et al., 2021). Our study site is located in Central Asia, which experiences an arid, semi-desert climate, and this type of climate is highly suitable for the growth of thick-walled fungi. Soil potassium (K) is the main factor affecting the distribution of Firmicutes (Vollú et al., 2014). Our correlation analysis confirmed this expectation, as K was significantly correlated with the abundance of Firmicutes in rhizosphere soil (Figure 6).

There were significant differences in the structure of the soil bacteria communities between rhizosphere soil and bulk soil under biochar application. Previous studies have shown that Firmicutes comprises a large portion of the bacterial community in rhizosphere soil (Teixeira et al., 2010; Ramos et al., 2019). However, we found that the abundance of Firmicutes was higher in bulk soil (40.34–45.25%) than in rhizosphere soil (20.53–42.09%; Figure 4). Firmicutes are known to generate desiccation-resistant endospores (Heulin et al., 2012; Ramos et al., 2019). Thus, we suspect that the high relative abundance of Firmicutes in bulk soil might stem from the production of large amounts of bacilli by members of the genus *Bacillus*, which have been shown to comprise approximately 21.41% of all bulk soil bacteria under unfavorable conditions (Song et al., 2013). Alternatively, the difference in the structure of the bacterial communities between rhizosphere soil and bulk soil might be explained by root exudates (Harel et al., 2012; Kolton et al., 2016; Coskun et al., 2017; Jaiswal et al., 2018b). Root exudates are a key source of nutrients for rhizosphere bacteria and have a substantial effect on the structure of soil microbial communities (Gu et al., 2020). We suggest that the application of biochar might alter the structure of the microbial communities of rhizosphere soil by increasing root-produced secretions and providing nutrients and energy that aid microbial metabolism and growth (Ma et al., 2019); the application of biochar could also contribute to differences in the structure of the bacteria communities in rhizosphere soil and bulk soil.

### Effect of biochar application on soil microbial communities

Our findings revealed that the application of biochar resulted in a significant increase in the α-diversity of bacterial communities in rhizosphere soil (Figure 3B). These findings are consistent with the results of previous studies showing that the application of biochar can lead to significant increases in the diversity of rhizosphere soil bacteria (Graber et al., 2010). The application of biochar has also been shown to significantly increase the α-diversity of bacterial communities in the rhizosphere soil of apple trees (Cao et al., 2021). Increases in soil nutrients might alter the structure and diversity of soil microbial communities (Hamer et al., 2004; Lehmann et al., 2011). We found that the application of biochar significantly increased the content of nutrients in rhizosphere soil (Supplementary Table S2), and this likely affects the diversity of the bacterial communities in rhizosphere soil (Toyama et al., 2011).In rhizosphere soil bacterial communities, the relative abundance of Firmicutes was significantly increased, and the abundance of Actinobacteria and Acidobacteria were decreased when biochar was applied at rates of 15.75–126.00 Mg ha^−1^. They were identified as biochar decomposers (Khodadad et al., 2011; Pezzolla et al., 2015). Firmicutes are fast-growing copiotrophs, and biochar provides nutrients (Fierer et al., 2007) and growth sites (Li et al., 2022), enhancing their competitiveness in the bacterial colonies. The relative abundance of soil acidobacteria was negatively correlated with soil pH, and the abundance of Acidobacteria decreased with the increase of pH (Mao et al., 2012; Männistö et al., 2007; Lauber et al., 2008; Chu et al., 2010; Griffiths et al., 2011). However, there was no significant correlation between the relative abundance of Acidobacteria and soil pH values in this study (Figure 6). This may be influenced by other environmental factors in the soil (Navarrete et al., 2013). The abundance of Acidobacteria decreased may be caused by different Acidobacteria subgroups, or even different Acidobacteria bacteria in the same subgroup, which have different responses to soil environmental factors (Jones et al., 2009; Zhang et al., 2014). The application of willow branch biochar (17.00–68.00 Mg ha^−1^) has been shown to increase the relative abundance of Actinobacteria (Prayogo et al., 2014); however, we found that biochar application resulted in a decrease in the abundance of Actinobacteria. This might stem from differences in the type of biochar applied (Wheat straw was the source of the biochar used in this experiment). The application of biochar does not appear to affect the growth of Actinobacteria in soil over short periods; however, the abundance of Actinobacteria tends to increase in the long term (Xu et al., 2020). This finding indicates that the effect of biochar application on microorganisms varies depending on the length of time since biochar application. In addition, the higher amount of available C in the biochar used in the present study, may explain the decrease in Actinobacteria in biochar, whose abundance was supposed to be associated with the degradation of recalcitrant carbon compounds (Bai et al., 2020).

The structure of microbial communities has also been shown to vary with habitat type and crop type (Kolton et al., 2011), suggesting that habitat type and crop type can have substantial effects on microbial communities (Kolton et al., 2016). We found that the application of biochar (15.75–31.50 Mg ha^−1^) resulted in significant decreases in the abundance of Proteobacteria and Alphaproteobacteria in the rhizosphere soil. As previous studies have shown that the abundance of Proteobacteria is higher in soils with high C availability (Fierer et al., 2007), it not consistent with our results, we found that application of biochar reduced the decline in relative abundance of proteobacteria. Firstly, our study found that there was no significant correlation between the abundance of Proteobacteria and SOC, but was positively correlated with AN, AP, and AK in the rhizosphere soil (Figure 6), it consistent with previous studies (Dai et al., 2018). It may be that the changes in other soil nutrients mask the role of SOC. Secondly, some scholars found that the abundance change of Firmicutes was completely opposite to that of Proteobacteria (Li et al., 2020b). Although biochar application improved soil nutrients to some extent, other microorganisms showed more competitiveness in this process (e.g., Firmicutes in this study; Gregory et al., 2015; Herrmann et al., 2019). Finally, different from other studies, our study was carried out in the seventh year after biochar application, so it may have different effects on Proteobacteria.

### The role of core soil microorganisms in maize root growth

We identified the core microbial taxa in the soil by constructing soil microbial co-occurrence networks (Figure 7). We found that there were stronger interactions among rhizosphere soil microorganisms than among bulk soil microorganisms. This might stem from the fact that rhizosphere soil is richer in nutrients than bulk soil (Supplementary Table S2); consequently, competitively superior taxa become dominant, and over time this can lead to the establishment of an equilibrium among dominant taxa (Nielsen et al., 2014; Li et al., 2021). We also found that core microbes were closely related to the growth of maize roots (Figure 8). This might stem from the role of core microbiota in promoting nutrient uptake by maize. Given that the content of nutrients accessible to maize in aeolian sandy soil is low, core microorganisms facilitate the uptake of nutrients by maize roots, which promote root growth (Yeoh et al., 2016). Analysis of the composition of core microorganisms revealed that the fungal phyla Basidiomycota and Ascomycota and the bacterial phylum Firmicutes play key roles in the growth of maize roots (Supplementary Figure S3). Previous studies have shown that the abundance of Ascomycota is affected by soil properties, as ascomycete fungi decompose organic matter around plant roots and promote their growth (Bastida et al., 2013). Basidiomycete fungi can decompose complex organic compounds in the soil; they thus play a key role in the formation of humus in the soil, and their activity promotes the growth of plant roots (Kjøller and Rosendahl, 2014). Previous studies of lemon rhizosphere soil have shown that *Bacillus cereus*, *Bacillus simplex*, and *Bacillus* sp. (all of which are thick-walled bacteria) promote the growth of primary roots and lateral roots, and this effect was achieved through the release of volatile organic compounds that altered the architecture of the root system (Egidi et al., 2019). These findings are consistent with the results of our study.

In summary, analyzed in relation to root growth and soil physicochemical properties, the application of 126.00 Mg hm^−2^ biochar had the best promotion effect on maize root growth after 7 years of biochar application. Biochar application changed maize root architecture by affecting soil physical properties, chemical properties, and soil microbial communities. Biochar application significantly altered soil moisture content, bulk density, and nutrient content, and can directly promote plant root growth (Abiven et al., 2015). Application of biochar affected the growth, development, and metabolism of soil bacteria by altering soil physicochemical properties (Zhu et al., 2017; Siedt et al., 2021). It increases the diversity of the rhizosphere soil bacterial community and changes the microbial structure (Ding et al., 2016), which in turn can maintain plant root growth (Bourceret et al., 2022). At the same time, core microorganisms play a key role in promoting nutrient uptake and root growth in the maize root system (Yeoh et al., 2016). Therefore, future research needs to pay more attention to the long-term effects of multiple factors on the architecture of maize roots.

## Conclusion

Our study showed that 7 years after application of biochar significantly promoted the growth of maize roots, with the best effect when biochar was applied at 126.00 Mg ha^−1^ and biochar application had a major effect on the bacterial communities in rhizosphere soil. The microbial communities of rhizosphere soil and bulk soil significantly differed. The bacterial communities in rhizosphere soil and core microorganisms play key roles in shaping the architecture of the maize root system. These findings enhance our understanding of the relationships between the architecture of maize roots and microorganisms in aeolian sandy soils. Additional studies are needed to characterize changes in root architecture and the soil microbial community during the entire growth period of maize through long-term field experiments.

## Data availability statement

The datasets presented in this study can be found in online repositories. The names of the repository/repositories and accession number(s) can be found at: https://www.ncbi.nlm.nih.gov/, PRJNA855115.

## Author contributions

HY, YH, MC, and XS conceived and designed the study and wrote the manuscript. HY, YH, MC, CQ, ZY, and HJ were responsible for performing the field and laboratory work. HY, YH, XS, and HJ analyzed the data. ZY, XZ, WX, and GT helped to perform the analysis with constructive discussions. All authors contributed to the article and approved the submitted version.

## Funding

This study was supported by the National Key Research and Development Program of China (2021YFD1900802), the Natural Science Foundation of Xinjiang Uygur Autonomous Region (2021D01A88), and the Open Fund of Key Laboratory of Agricultural Environment of Northwest Oasis, Ministry of Agriculture and Rural Affairs, China (XBLZ-202004).

## Conflict of interest

The authors declare that the research was conducted in the absence of any commercial or financial relationships that could be construed as a potential conflict of interest.

## Publisher’s note

All claims expressed in this article are solely those of the authors and do not necessarily represent those of their affiliated organizations, or those of the publisher, the editors and the reviewers. Any product that may be evaluated in this article, or claim that may be made by its manufacturer, is not guaranteed or endorsed by the publisher.

## Footnotes

^1^ http://huttenhower.sph.harvard.edu/lefse/

## References

[ref1] Abiven S. Hund A. Martinsen V. Cornelissen G. (2015). Biochar amendment increases maize root surface areas and branching: a shovelomics study in Zambia. Plant Soil 395, 45–55. doi: 10.1007/s11104-015-2533-2

[ref2] Akhil D. Lakshmi D. Kartik A. Vo D. N. Arun J. Gopinath K. P. (2021). Production, characterization, activation and environmental applications of engineered biochar: a review. Environ. Chem. Lett. 19, 2261–2297. doi: 10.1007/s10311-020-01167-7

[ref3] Anderson C. R. Condron L. M. Clough T. J. Fiers M. Stewart A. Hill R. A.. (2011). Biochar induced soil microbial community change: implications for biogeochemical cycling of carbon, nitrogen and phosphorus. Pedobiologia 54, 309–320. doi: 10.1016/j.pedobi.2011.07.005

[ref4] Baccile N. Laurent G. Babonneau F. Fayon F. Titirici M. M. Antonietti M. (2009). Structural characterization of hydrothermal carbon spheres by advanced solid-state MAS13C NMR investigations. J. Phys. Chem. C 113, 9644–9654. doi: 10.1021/jp901582x

[ref5] Bai N. Zhang H. Zhou S. Sun H. Zhao Y. Zheng X.. (2020). Long-term effects of straw return and straw-derived biochar amendment on bacterial communities in soil aggregates. Sci. Rep. 10, 7891–7810. doi: 10.1038/s41598-020-64857-w, PMID: 32398757 PMC7217948

[ref6] Bastida F. Hernández T. Albaladejo J. García C. (2013). Phylogenetic and functional changes in the microbial community of long-term restored soils under semiarid climate. Soil Biol. Biochem. 65, 12–21. doi: 10.1016/j.soilbio.2013.04.022

[ref7] Bengough A. G. Young I. M. (1993). Root elongation of seedling peas through layered soil of different penetration resistances. Plant Soil 149, 129–139. doi: 10.1007/BF00010770

[ref8] Bourceret A. Guan R. Dorau K. Mansfeldt T. Omidbakhshfard A. Medeiros D. B.. (2022). Maize field study reveals covaried microbiota and metabolic changes in roots over plant growth. MBio 13, e02584–e02521. doi: 10.1128/mbio.02584-21, PMID: 35258335 PMC9040757

[ref9] Busschei W. J. Novak J. M. Evans D. E. Watts D. W. Niandou M. A. S. Ahmedna M. (2010). Influence of pecan biochar on physical properties of a Norfork loamy sand. Soil Sci. 175, 10–14. doi: 10.1097/ss.0b013e3181cb7f46

[ref10] Cao H. Jia M. Xun M. Wang X. Chen K. Yang H. (2021). Nitrogen transformation and microbial community structure varied in apple rhizosphere and rhizoplane soils under biochar amendment. J. Soils Sediments 21, 853–868. doi: 10.1007/s11368-020-02868-w

[ref11] Carvalho M. T. M. Madari B. E. Bastiaans L. van Oort P. N. Leal W. G. O. Heinemann A. B.. (2016). Properties of a clay soil from 1.5 to 3.5 years after biochar application and the impact on rice yield. Geoderma 276, 7–18. doi: 10.1016/j.geoderma.2016.04.013

[ref12] Chen M. Zhu X. Zhao C. Yu P. Abulaizi M. Jia H. (2021). Rapid microbial community evolution in initial Carex litter decomposition stages in Bayinbuluk alpine wetland during the freeze–thaw period. Ecol. Indic. 121:107180. doi: 10.1016/j.ecolind.2020.107180

[ref13] Chu H. Fierer N. Lauber C. L. Caporaso J. G. Knight R. Grogan P. (2010). Soil bacterial diversity in the Arctic is not fundamentally different from that found in other biomes. Environ. Microbiol. 12, 2998–3006. doi: 10.1111/j.1462-2920.2010.02277.x 20561020

[ref14] Coskun D. Britto D. T. Shi W. Kronzucker H. J. (2017). How plant root exudates shape the nitrogen cycle. Trends Plant Sci. 22, 661–673. doi: 10.1016/j.tplants.2017.05.004, PMID: 28601419

[ref15] Crane-Droesch A. Abiven S. Jeffery S. Torn M. S. (2013). Heterogeneous global crop yield response to biochar: a meta-regression analysis. Environ. Res. Lett. 8:044049. doi: 10.1088/1748-9326/8/4/044049

[ref16] Dai Z. Su W. Chen H. Barberánc A. Zhao H. Yu M.. (2018). Long-term nitrogen fertilization decreases bacterial diversity and favors the growth of Actinobacteria and Proteobacteria in agro-ecosystems across the globe. Glob. Chang. Biol. 24, 3452–3461. doi: 10.1111/gcb.14163, PMID: 29645398

[ref17] Dardanelli J. L. Ritchie J. T. Calmon M. Andriani J. M. Collino D. J. (2004). An empirical model for root water uptake. Field Crop Res. 87, 59–71. doi: 10.1016/j.fcr.2003.09.008

[ref18] de Vries F. T. Griffiths R. I. Bailey M. Craig H. Girlands M. Gweon H. S.. (2018). Soil bacterial networks are less stable under drought than fungal networks. Nat. Commun. 9:3033. doi: 10.1038/s41467-018-05516-7, PMID: 30072764 PMC6072794

[ref19] Dempster D. N. Gleeson D. B. Solaiman Z. M. Jones D. L. Murphy D. V. (2011). Decreased soil microbial biomass and nitrogen mineralisation with eucalyptus biochar addition to a coarse textured soil. Plant Soil 354, 311–324. doi: 10.1007/s11104-011-1067-5

[ref20] Ding Y. Liu Y. Liu S. Li Z. Tan X. Huang X.. (2016). Biochar to improve soil fertility. A review. Agron. Sustain. Dev. 36:36. doi: 10.1007/s13593-016-0372-z

[ref21] Driessen P. Deckers J. Spaargaren O. Nachtergaele F. O. (2001). Lecture Notes on the Major Soils of the World. World soil resources reports. ISSN:0532–0488

[ref22] Egidi E. Delgado-Baquerizo M. Plett J. M. Wang J. Eldridge D. J. Bardgett R. D.. (2019). A few Ascomycota taxa dominate soil fungal communities worldwide. Nat. Commun. 10:2369. doi: 10.1038/s41467-019-10373-z, PMID: 31147554 PMC6542806

[ref23] Feng W. Yang F. Cen R. Liu J. Qu Z. Miao Q.. (2021). Effects of straw biochar application on soil temperature, available nitrogen and growth of corn. J. Environ. Manag. 277:111331. doi: 10.1016/j.jenvman.2020.111331, PMID: 32949951

[ref24] Fierer N. Bradford M. A. Jackson R. B. (2007). Toward an ecological classification of soil bacteria. Ecology 88, 1354–1364. doi: 10.1890/05-1839 17601128

[ref25] Gao H. He X. Geng Z. She D. Yin J. (2011). Effects of biochar and biochar-based nitrogen fertilizer on soil water-holding capacity. Chin. Agric. Sci. Bull. 27, 207–213.

[ref26] Ge X. Li Y. Luloff A. E. Dong K. Xiao J. (2015). Effect of agricultural economic growth on sandy desertification in Horqin Sandy land. Ecol. Econ. 119, 53–63. doi: 10.1016/j.ecolecon.2015.08.006

[ref27] Glab T. Zabinski A. Sadowska U. Gondek K. Kopec M. Mierzwa-Hersztek M.. (2018). Effects of co-composted maize, sewage sludge, and biochar mixtures on hydrological and physical qualities of sandy soil. Geoderma 315, 27–35. doi: 10.1016/j.geoderma.2017.11.034

[ref28] Glaser B. Lehmann J. Zech W. (2002). Ameliorating physical and chemical properties of highly weathered soils in the tropics with charcoal–a review. Biol. Fertil. Soils 35, 219–230. doi: 10.1007/s00374-002-0466-4

[ref29] Goodman A. M. Ennos A. R. (1999). The effects of soil bulk density on the morphology and anchorage mechanics of the root systems of sunflower and maize. Ann. Bot. 83, 293–302. doi: 10.1006/anbo.1998.0822

[ref30] Graber E. R. Harel Y. M. Kolton M. Cytryn E. Silber A. David D. R.. (2010). Biochar impact on development and productivity of pepper and tomato grown in fertigated soilless media. Plant Soil 337, 481–496. doi: 10.1007/s11104-010-0544-6

[ref31] Gregory S. J. Anderson C. W. N. Camps-Arbestain M. Biggs P. J. Ganley A. R. D. O’Sullivan J. M.. (2015). Biochar in co-contaminated soil manipulates arsenic solubility and microbiological community structure, and promotes organochlorine degradation. PLoS One 10:0125393. doi: 10.1371/journal.pone.0125393, PMID: 25923541 PMC4414470

[ref32] Griffiths R. I. Thomson B. C. James P. Bell T. Bailey M. Whiteley A. S. (2011). The bacterial biogeography of British soils. Environ. Microbiol. 13, 1642–1654. doi: 10.1111/j.1462-2920.2011.02480.x, PMID: 21507180

[ref33] Gu Y. Wang X. Yang T. Friman V. Geisen S. Wei Z.. (2020). Chemical structure predicts the effect of plant-derived low-molecular weight compounds on soil microbiome structure and pathogen suppression. Funct. Ecol. 34, 2158–2169. doi: 10.1111/1365-2435.13624

[ref34] Guimerà R. Amaral L. A. N. (2005). Functional cartography of complex metabolic networks. Nature 433, 895–900. doi: 10.1038/nature03288, PMID: 15729348 PMC2175124

[ref35] Hamer U. Marschner B. Brodowski S. Amelung W. (2004). Interactive priming of black carbon and glucose mineralisation. Org. Geochem. 35, 823–830. doi: 10.1016/j.orggeochem.2004.03.003

[ref36] Han X. Zhao Y. Gao X. Jiang S. Lin L. An T. (2021). Virtual water output intensifies the water scarcity in Northwest China: current situation, problem analysis and countermeasures. Sci. Total Environ. 765:144276. doi: 10.1016/j.scitotenv.2020.144276, PMID: 33401056

[ref37] Harel Y. M. Elad Y. Rav-David D. Borenstein M. Shulchani R. Lew B.. (2012). Biochar mediates systemic response of strawberry to foliar fungal pathogens. Plant Soil 357, 245–257. doi: 10.1007/s11104-012-1129-3

[ref38] Hayward A. C. Fegan N. Fegan M. Stirling G. R. (2010). Stenotrophomonas and Lysobacter: ubiquitous plant-associated gamma-proteobacteria of developing significance in applied microbiology. J. Appl. Microbiol. 108, 756–770. doi: 10.1111/j.1365-2672.2009.04471.x, PMID: 19702860

[ref39] Herrmann L. Lesueur D. Robin A. Robain H. Wiriyakitnateekul W. Brtäu L. (2019). Impact of biochar application dose on soil microbial communities associated with rubber trees in north East Thailand. Sci. Total Environ. 689, 970–979. doi: 10.1016/j.scitotenv.2019.06.441, PMID: 31280178

[ref40] Heulin T. Luca G. D. Barakat M. Groot A. D. Blanchard L. Ortet P.. (2012). Bacterial adaptation to hot and dry deserts. Adapt. Microb. Life Environ. Extrem. 4, 69-85. doi: 10.1007/978-3-211-99691-1_4

[ref41] Hodge A. (2004). The plastic plant: root responses to heterogeneous supplies of nutrients. New Phytol. 162, 9–24. doi: 10.1111/j.1469-8137.2004.01015.x

[ref42] Hu Y. Chen M. Yang Z. Cong M. Zhu X. Jia H. (2022). Soil microbial community response to nitrogen application on a swamp meadow in the arid region of Central Asia. Front. Microbiol. 12:797306. doi: 10.3389/fmicb.2021.797306, PMID: 35126333 PMC8811146

[ref43] Hu L. Li S. Li K. Huang H. Wan W. Huang Q.. (2020). Effects of two types of straw biochar on the mineralization of soil organic carbon in farmland. Sustain. For. 12, 1–18. doi: 10.3390/SU122410586

[ref44] Jaiswal A. K. Elad Y. Cytryn E. Graber E. R. Frenkel O. (2018a). Activating biochar by manipulating the bacterial and fungal microbiome through pre-conditioning. New Phytol. 219, 363–377. doi: 10.1111/nph.15042, PMID: 29417582

[ref45] Jaiswal A. K. Frenkel O. Tsechansky L. Elad Y. Graber E. R. (2018b). Immobilization and deactivation of pathogenic enzymes and toxic metabolites by biochar: a possible mechanism involved in soilborne disease suppression. Soil Biol. Biochem. 121, 59–66. doi: 10.1016/j.soilbio.2018.03.001

[ref46] Jones R. T. Robeson M. S. Lauber C. L. Hamady M. Knight R. Fierer N. (2009). A comprehensive survey of soil acidobacterial diversity using pyrosequencing and clone library analyses. ISME J. 3, 442–453. doi: 10.1038/ismej.2008.127, PMID: 19129864 PMC2997719

[ref47] Kari A. Nagymáté Z. Romsics C. Vajna B. Tóth E. Lazanyi-Kovács R.. (2021). Evaluating the combined effect of biochar and PGPR inoculants on the bacterial community in acidic sandy soil. Appl. Soil Ecol. 160:103856. doi: 10.1016/j.apsoil.2020.103856

[ref48] Khodadad C. L. M. Zimmerman A. R. Green S. J. Uthandi S. Foster J. S. (2011). Taxa-specific changes in soil microbial community composition induced by pyrogenic carbon amendments. Soil Biol. Biochem. 43, 385–392. doi: 10.1016/j.soilbio.2010.11.005

[ref49] Kimetu J. M. Lehmann J. (2010). Stability and stabilisation of biochar and green manure in soil with different organic carbon contents. Aust. J. Soil Res. 48, 577–585. doi: 10.1071/SR10036

[ref50] Kjøller R. Rosendahl S. (2014). Cultivated and fallow fields harbor distinct communities of basidiomycota. Fungal Ecol. 9, 43–51. doi: 10.1016/j.funeco.2014.02.005

[ref51] Kolb S. E. Fermanich K. J. Dornbush M. E. (2009). Effect of charcoal quantity on microbial biomass and activity in temperate soils. Soil Sci. Soc. Am. J. 73, 1173–1181. doi: 10.2136/sssaj2008.0232

[ref52] Kõljalg U. Nilsson R. H. Abarenkov K. Tedersoo L. Taylor A. F. Bahram M.. (2013). Towards a unified paradigm for sequence-based identification of fungi. Mol. Ecol. 22, 5271–5277. doi: 10.1111/mec.12481, PMID: 24112409

[ref53] Kolton M. Graber E. R. Tsehansky L. Elad Y. Cytryn E. (2016). Biochar-stimulated plant performance is strongly linked to microbial diversity and metabolic potential in the rhizosphere. New Phytol. 213, 1393–1404. doi: 10.1111/nph.14253, PMID: 27780299

[ref54] Kolton M. Harel Y. M. Pasternak Z. Graber E. R. Elad Y. Cytryn E. (2011). Impact of biochar application to soil on the root-associated bacterial community structure of fully developed greenhouse pepper plants. Appl. Environ. Microbiol. 77, 4924–4930. doi: 10.1128/aem.00148-11, PMID: 21622786 PMC3147372

[ref55] Kwak M. Kong H. G. Choi K. Kwon S. Song J. Y. Lee J.. (2018). Rhizosphere microbiome structure alters to enable wilt resistance in tomato. Nat. Biotechnol. 36, 1100–1109. doi: 10.1038/nbt.4232, PMID: 30295674

[ref56] Laird D. A. Fleming P. Davis D. D. Horton R. Wang B. Karlen D. L. (2010). Impact of biochar amendments on the quality of a typical Midwestern agricultural soil. Geoderma 158, 443–449. doi: 10.1016/j.geoderma.2010.05.013

[ref57] Lal R. (2005). World crop residues production and implications of its use as a biofuel. Environ. Int. 31, 575–584. doi: 10.1016/j.envint.2004.09.005, PMID: 15788197

[ref58] Langmann B. Duncan B. Textor C. Trentmann J. van der Werf G. R. (2009). Vegetation fire emissions and their impact on air pollution and climate. Atmos. Environ. 43, 107–116. doi: 10.1016/j.atmosenv.2008.09.047

[ref59] Lauber C. L. Strickland M. S. Bradford M. A. Fierer N. (2008). The influence of soil properties on the structure of bacterial and fungal communities across land-use types. Soil Biol. Biochem. 40, 2407–2415. doi: 10.1016/j.soilbio.2008.05.021

[ref60] Lehmann J. Gaunt J. Rondon M. (2006). Bio-char sequestration in terrestrial ecosystems–a review. Mitig. Adapt. Strateg. Glob. Chang. 11, 403–427. doi: 10.1007/s11027-005-9006-5

[ref61] Lehmann J. Rillig M. C. Thies J. Masiello C. A. Hockaday W. C. Crowley D. (2011). Biochar effects on soil biota - a review. Soil Biol. Biochem. 43, 1812–1836. doi: 10.1016/j.soilbio.2011.04.022

[ref62] Li E. Jonge R. D. Liu C. Jiang H. Friman V. Pieterse C. M. J.. (2021). Rapid evolution of bacterial mutualism in the plant rhizosphere. Nat. Commun. 12:3829. doi: 10.1038/s41467-021-24005-y, PMID: 34158504 PMC8219802

[ref63] Li X. Song Y. Bian Y. Gu C. Yang X. Wang F.. (2020a). Insights into the mechanisms underlying efficient Rhizodegradation of PAHS in biochar-amended soil: from microbial communities to soil metabolomics. Environ. Int. 144:105995. doi: 10.1016/j.envint.2020.105995, PMID: 32758715

[ref64] Li X. Wu M. Xue Y. (2022). Nickel-loaded shrimp shell biochar enhances batch anaerobic digestion of food waste. Bioresour. Technol. 352:127092. doi: 10.1016/j.biortech.2022.127092, PMID: 35367323

[ref65] Li W. Zhang Y. Mao W. Wang C. Yin S. (2020b). Functional potential differences between Firmicutes and Proteobacteria in response to manure amendment in a reclaimed soil. Can. J. Microbiol. 66, 689–697. doi: 10.1139/cjm-2020-0143, PMID: 32717168

[ref66] Liang B. Lehmann J. Solomon D. Kinyangi J. Grossman J. O’Neill B.. (2006). Black carbon increases cation exchange capacity in soils. Soil Sci. Soc. Am. J. 70, 1719–1730. doi: 10.2136/sssaj2005.0383

[ref67] Lu S. Sun F. Zong Y. (2014). Effect ofrice husk biochar and coal fly ash on some physical properties of expansive clayey soil (vertisol). Catena 114, 37–44. doi: 10.1016/j.catena.2013.10.014

[ref68] Luo S. Wang S. Tian L. Li S. Li X. Shen Y.. (2017). Long-term biochar application influences soil microbial community and its potential roles in semiarid farmland. Appl. Soil Ecol. 117-118, 10–15. doi: 10.1016/j.apsoil.2017.04.024

[ref69] Lynch J. P. (2011). Root phenes for enhanced soil exploration and phosphorus acquisition: tools for future crops. Plant Physiol. 156, 1041–1049. doi: 10.1104/pp.111.175414, PMID: 21610180 PMC3135935

[ref70] Ma W. (2021). Effect of aging biochar on organic carbon fractions and carbon emission of Sandy soil in the arid region. Master of science. Xinjiang Agricultural University. Urumqi, Xinjiang Uygur Autonomous Region, China. 12–13.

[ref71] Ma B. Huang R. Zhang N. Su B. Liang Y. (2019). Effect of straw-derived biochar on molecular ecological network between bacterial and fungal communities in rhizosphere soil. Acta Pedol. Sin. 56, 964–974. doi: 10.11766/trxb201809030443

[ref72] Männistö M. K. Tiirola M. Häggblom M. M. (2007). Bacterial communities in Arctic fjelds of Finnish Lapland are stable but highly pH-dependent. FEMS Microbiol. Ecol. 59, 452–465. doi: 10.1111/j.1574-6941.2006.00232.x, PMID: 17328122

[ref73] Mao J. D. Johnson R. L. Lehmann J. Olk D. C. Neves E. G. Thompson M. L.. (2012). Abundant and stable char residues in soils: implications for soil fertility and carbon sequestration. Environ. Sci. Technol. 46, 9571–9576. doi: 10.1021/es301107c, PMID: 22834642

[ref74] Mei N. Zhang X. Wang X. Peng C. Gao H. Zhu P.. (2021). Effects of 40 years applications of inorganic and organic fertilization on soil bacterial community in a maize agroecosystem in Northeast China. Eur. J. Agron. 130:126332. doi: 10.1016/J.EJA.2021.126332

[ref75] Navarrete A. A. Kuramae E. E. d. Hollander M. Pijl A. S. van Veen J. A. Tsai S. M. (2013). Acidobacterial community responses to agricultural management of soybean in Amazon forest soils. FEMS Microbiol. Ecol. 83, 607–621. doi: 10.1111/1574-6941.12018, PMID: 23013447

[ref76] Nazih N. Finlay-Moore O. Hartel P. G. Fuhrmann J. J. (2001). Whole soil fatty acid methyl ester (FAME) profiles of early soybean rhizosphere as affected by temperature and matric water potential. Soil Biol. Biochem. 33, 693–696. doi: 10.1016/S0038-0717(00)00197-8

[ref77] Nelissen V. Ruysschaert G. Manka’Abusi D. D’Hose T. Beuf K. D. Al-Barri B.. (2015). Impact of a woody biochar on properties of a sandy loam soil and spring barley during a two-year field experiment. Eur. J. Agron. 62, 65–78. doi: 10.1016/j.eja.2014.09.006

[ref78] Nielsen S. Minchin T. Kimber S. Zwieten L. V. Gilbert J. Munroe P.. (2014). Comparative analysis of the microbial communities in agricultural soil amended with enhanced biochars or traditional fertilisers. Agric. Ecosyst. Environ. 191, 73–82. doi: 10.1016/j.agee.2014.04.006

[ref79] Novak J. M. Busscher W. J. Laird D. L. Ahmedna M. Watts D. W. Niandou M. A. S. (2009). Impact of biochar amendment on fertility of a southeastern coastal plain soil. Soil Sci. 174, 105–112. doi: 10.1097/SS.0b013e3181981d9a

[ref80] Olmo M. Villar R. Salazar P. Alburquerque J. (2016). Changes in soil nutrient availability explain biochar’s impact on wheat root development. Plant Soil 399, 333–343. doi: 10.1007/s11104-015-2700-5

[ref81] Peng Y. Niu J. Peng Z. Zhang F. Zhang F. Li C. (2010). Shoot growth potential drives n uptake in maize plants and correlates with root growth in the soil. Field Crop Res. 115, 85–93. doi: 10.1016/j.fcr.2009.10.006

[ref82] Pezzolla D. Marconi G. Turchetti B. Zadra C. Agnelli A. Veronesi F.. (2015). Influence of exogenous organic matter on prokaryotic and eukaryotic microbiota in an agricultural soil. A multidisciplinary approach. Soil Biol. Biochem. 82, 9–20. doi: 10.1016/j.soilbio.2014.12.008

[ref83] Pietikäinen J. Kikilä O. Fitze H. (2000). Charcoal as a habitat for microbes and its effect on the microbial community of the underlying humus. Oikos 89, 231–242. doi: 10.1034/j.1600-0706.2000.890203.x

[ref84] Poudel R. Jumpponen A. Schlatter D. C. Paulitz T. C. Gardener B. M. Kinkel L. L.. (2016). Microbiome networks: a systems framework for identifying candidate microbial assemblages for disease management. Phytopathology 106, 1083–1096. doi: 10.1094/PHYTO-02-16-0058-FI, PMID: 27482625

[ref85] Prayogo C. Jones J. E. Baeyens J. Bending G. D. (2014). Impact of biochar on mineralisation of C and N from soil and willow litter and its relationship with microbial community biomass and structure. Biol. Fertil. Soils 50, 695–702. doi: 10.1007/s00374-013-0884-5

[ref86] Quast C. Pruesse E. Yilmaz P. Gerken J. Schweer T. Yarza P.. (2013). The SILVA ribosomal RNA gene database project: improved data processing and web-based tools. Nucleic Acids Res. 41, D590–D596. doi: 10.1093/nar/gks1219, PMID: 23193283 PMC3531112

[ref87] Ramos L. R. Vollú R. E. Jurelevicius D. Rosado A. S. Seldin L. (2019). Firmicutes in different soils of Admiralty Bay, King George Island. Polar Biol. 42, 2219–2226. doi: 10.1007/s00300-019-02596-z

[ref88] Schmidt H. Eickhorst T. (2014). Detection and quantification of native microbial populations on soil-grown rice roots by catalyzed reporter deposition-fluorescence in situ hybridization. FEMS Microbiol. Ecol. 87, 390–402. doi: 10.1111/1574-6941.12232, PMID: 24118011

[ref89] Schmidt H. Nunan N. Höck A. Eickhorst T. Kaiser C. Woebken D.. (2018). Recognizing patterns: spatial analysis of observed microbial colonization on root surfaces. Front. Environ. Sci. 6:61. doi: 10.3389/fenvs.2018.00061

[ref90] Shao K. Bai C. Cai J. Hu Y. Gong Y. Chao J.. (2019). Illumina sequencing revealed soil microbial communities in a Chinese alpine grassland. Geomicrobiol J. 36, 204–211. doi: 10.1080/01490451.2018.1534902

[ref91] Siedt M. Schäffer A. Smith K. E. C. Nabel M. Roß-Nickoll M. Dongen J. T. V. (2021). Comparing straw, compost, and biochar regarding their suitability as agricultural soil amendments to affect soil structure, nutrient leaching, microbial communities, and the fate of pesticides. Sci. Total Environ. 751, 141607–141628. doi: 10.1016/j.scitotenv.2020.141607, PMID: 32871314

[ref92] Sohi S. P. Krull E. Lopez-Capel E. Bol R. (2010). A review of biochar and its use and function in soil. Adv. Agron. 105, 47–82. doi: 10.1016/S0065-2113(10)05002-9

[ref93] Soinne H. Hovi J. Tammeorg P. Turtola E. (2014). Effect of biochar on phosphorus sorption and clay soil aggregate stability. Geoderma 219-220, 162–167. doi: 10.1016/j.geoderma.2013.12.022

[ref94] Song Z. Wang F. Zhi X. Chen J. Zhou E. Liang F.. (2013). Bacterial and archaeal diversities in Yunnan and Tibetan hot springs, China. Environ. Microbiol. 15, 1160–1175. doi: 10.1111/1462-2920.12025, PMID: 23126508

[ref95] Teixeira L. C. Peixoto R. S. Cury J. C. Sul W. J. Pellizari V. H. Tiedje J.. (2010). Bacterial diversity in rhizosphere soil from antarctic vascular plants of Admiralty Bay, maritime Antarctica. ISME J. 4, 989–1001. doi: 10.1038/ismej.2010.35, PMID: 20357834

[ref96] Todd-Brown K. E. O. Hopkins F. M. Kivlin S. N. Talbot J. M. Allison S. D. (2012). A framework for representing microbial decomposition in coupled climate models. Biogeochemistry 109, 19–33. doi: 10.1007/s10533-011-9635-6

[ref97] Toju H. Tanabe A. S. Sato H. (2018). Network hubs in root-associated fungal metacommunities. Microbiome 6:116. doi: 10.1186/s40168-018-0497-1, PMID: 29935536 PMC6015470

[ref98] Toyama T. Furukawa T. Maeda N. Inoue D. Sei K. Mori K.. (2011). Accelerated biodegradation of pyrene and benzo[a]pyrene in the Phragmites australis rhizosphere by bacteria-root exudate interactions. Water Res. 45, 1629–1638. doi: 10.1016/j.watres.2010.11.044, PMID: 21196023

[ref99] Trivedi P. Leach J. E. Tringe S. G. Sa T. Singh B. K. (2020). Plant–microbiome interactions: from community assembly to plant health. Nat. Rev. Microbiol. 18, 607–621. doi: 10.1038/s41579-020-0412-1, PMID: 32788714

[ref100] Vejan P. Abdullah R. Khadiran T. Ismail S. Boyce A. N. (2016). Role of plant growth promoting rhizobacteria in agricultural sustainability - a review. Molecules 21:573. doi: 10.3390/molecules21050573, PMID: 27136521 PMC6273255

[ref101] Vollú R. E. Jureleviciu D. Ramos L. R. Peixoto R. S. Rosado A. S. Seldin L. (2014). Aerobic endospore-forming bacteria isolated from Antarctic soils as producers of bioactive compounds of industrial interest. Polar Biol. 37, 1121–1131. doi: 10.1007/s00300-014-1505-y

[ref102] Wang G. Xu Y. Jin J. Liu J. Zhang Q. Liu X. (2009). Effect of soil type and soybean genotype on fungal community in soybean rhizosphere during reproductive growth stages. Plant Soil 317, 135–144. doi: 10.1007/s11104-008-9794-y

[ref103] Wardle D. A. Nilsson M. Zackrisson O. (2008). Fire-derived charcoal causes loss of forest humus. Science 320:629. doi: 10.1126/science.1154960, PMID: 18451294

[ref104] Wardle D. A. Zackrisson O. Nilsson M. (1998). The charcoal effect in boreal forests: mechanisms and ecological consequences. Oecologia 115, 419–426. doi: 10.1007/s004420050536, PMID: 28308435

[ref105] Xu W. Whitman W. B. Gundale M. J. Chien C. Chiu C. (2020). Functional response of the soil microbial community to biochar applications. GCB Bioenergy 13, 269–281. doi: 10.1111/gcbb.12773

[ref106] Yamato M. Okimori Y. Wibowo I. F. Anshori S. Ogawa M. (2006). Effects of the application of charred bark of Acacia mangium on the yield of maize, cowpea and peanut, and soil chemical properties in South Sumatra, Indonesia. Soil Sci. Plant Nutr. 52, 489–495. doi: 10.1111/j.1747-0765.2006.00065.x

[ref107] Yang Y. Wu P. (2020). Soil bacterial community varies but fungal community stabilizes along five vertical climate zones. Catena 195:104841. doi: 10.1016/j.catena.2020.104841

[ref108] Yao X. Min H. Lü Z. Yuan H. (2006). Influence of acetamiprid on soil enzymatic activities and respiration. Eur. J. Soil Biol. 42, 120–126. doi: 10.1016/j.ejsobi.2005.12.001

[ref109] Yeoh Y. K. Paungfoo-Lonhienne C. Dennis P. G. Robinson N. Ragan M. A. Schmidt S.. (2016). The core root microbiome of sugarcanes cultivated under varying nitrogen fertilizer application. Environ. Microbiol. 18, 1338–1351. doi: 10.1111/1462-2920.12925, PMID: 26032777

[ref110] Yin Q. Liu J. Liu G. Yang X. Li X. Zhang Y.. (2021). Effects of biochar application for four consecutive years on microbial community structure of tobacco cinnamon soil. J. Agric. Sci. Technol. 23, 176–185. doi: 10.13304/j.nykjdb.2019.0505

[ref111] Yu L. Yu M. Lu X. Tang C. Liu X. Brookes P. C.. (2018). Combined application of biochar and nitrogen fertilizer benefits nitrogen retention in the rhizosphere of soybean by increasing microbial biomass but not altering microbial community structure. Sci. Total Environ. 640-641, 1221–1230. doi: 10.1016/j.scitotenv.2018.06.018, PMID: 30021287

[ref112] Zhang Y. Cong J. Lu H. Li G. Qu Y. Su X.. (2014). Community structure and elevational diversity patterns of soil Acidobacteria. J. Environ. Sci. 26, 1717–1724. doi: 10.1016/j.jes.2014.06.012, PMID: 25108728

[ref113] Zhang C. Lin Y. Tian X. Xu Q. Chen Z. Lin W. (2017). Tobacco bacterial wilt suppression with biochar soil addition associates to improved soil physiochemical properties and increased rhizosphere bacteria abundance. Appl. Soil Ecol. 112, 90–96. doi: 10.1016/j.apsoil.2016.12.005

[ref114] Zhang Y. Tang G. Long X. Ge C. Xu W. (2021). Effects of one-time biochar input on soil properties and corn yield in irrigation sandy soil. Agric. Res. Arid Areas. 39, 137–141. doi: 10.7606/j.issn.1000-7601.2021.04.17

[ref115] Zhou H. Zhang D. Jiang Z. Sun P. Xiao H. Wu Y.. (2019). Changes in the soil microbial communities of alpine steppe at Qinghai-Tibetan plateau under different degradation levels. Sci. Total Environ. 651, 2281–2291. doi: 10.1016/j.scitotenv.2018.09.336, PMID: 30326458

[ref116] Zhu X. Chen B. Zhu L. Xing B. (2017). Effects and mechanisms of biochar-microbe interactions in soil improvement and pollution remediation: a review. Environ. Pollut. 227, 98–115. doi: 10.1016/j.envPol.2017.04.032, PMID: 28458251

