# Supplementary material for: Biochar-mediated changes in the microbial communities of rhizosphere soil alter the architecture of maize roots
Source: Front Microbiol. 2022 Oct 4;13:1023444. doi: 10.3389/fmicb.2022.1023444 (PMC9577002; doi:10.3389/fmicb.2022.1023444)
Supplement: Supplementary file 1 [file Data_Sheet_1.PDF]

ADDITIONAL FILES

TABLE S1 | Effect of biochar addition on the physical properties of bulk soil

Trtatment	Soil moisture content (%)	Saturated water content (%)	Field capacity (%)	Bulk density (g/cm^3)
CK	4.41±0.21a	30.73±0.73a	23.78±0.21a	1.453±0.042a
C1	4.38±0.42a	30.81±0.62a	23.95±0.09a	1.448±0.041b
C2	4.43±0.12a	30.81±0.73a	24.05±0.21a	1.432±0.012c
C3	4.41±0.21a	30.76±0.88a	23.94±0.32a	1.423±0.022d
C4	4.27±0.19b	30.78±0.57a	24.06±0.22a	1.419±0.031d

Different lowercase letters indicate significant differences among biochar application treatments ($P < 0.05$).

TABLE S2 | Effect of biochar addition on the chemical properties of rhizosphere soil and bulk soil

	Trtatment	pH	SOC (g/kg)	AN (mg/kg)	AP (mg/kg)	AK (mg/kg)	TN (g/kg)	TP (g/kg)	TK (g/kg)
Bulk	CK	8.19±0.22a	8.40±1.19ab	5.31±0.34d	12.38±1.52c	110.50±4.76c	4.60±0.35a	0.28±0.01c	2.73±0.11bc
	C1	8.21±0.18a	9.33±0.74ab	7.26±0.36c	12.30±1.57c	112.33±4.03c	4.17±0.28a	0.28±0.01bc	2.81±0.2bc
	C2	8.35±0.15a	7.62±0.59b	8.81±0.28b	13.51±0.50bc	128.00±3.03b	3.86±0.70a	0.29±0.01bc	2.85±0.19bc
	C3	8.25±0.24a	8.09±0.71b	12.68±0.57a	13.94±1.19b	130.17±2.48b	4.32±0.45a	0.30±0.05ab	3.04±0.55ab
	C4	8.23±0.07a	9.90±2.00a	12.95±0.44a	17.58±1.14a	142.50±5.99a	4.23±0.48a	0.32±0.01a	3.30±0.22a
Rhizosphere	CK	7.98±0.17a	10.65±1.68c	6.08±0.43e	14.08±0.59e	119.50±6.66d	4.16±0.05a	0.31±0.01c	2.91±0.32c
	C1	8.01±0.20a	11.66±0.80bc	6.72±0.50d	15.22±1.19d	135.17±1.72c	4.56±0.08a	0.33±0.01b	3.30±0.28ab
	C2	8.06±0.28a	11.59±0.57bc	8.89±0.57c	16.79±1.22c	142.40±2.58b	4.13±0.40a	0.33±0.01b	3.35±0.11ab
	C3	7.87±0.20a	12.46±0.71ab	9.48±0.49b	17.93±0.45b	141.33±6.09b	4.31±0.21a	0.36±0.01a	3.23±0.11b
	C4	7.96±0.22a	13.19±1.03a	12.56±0.42a	19.14±0.84a	183.17±2.93a	4.31±0.39a	0.37±0.01a	3.53±0.24a

Different lowercase letters indicate significant differences among biochar application treatments (P < 0.05).

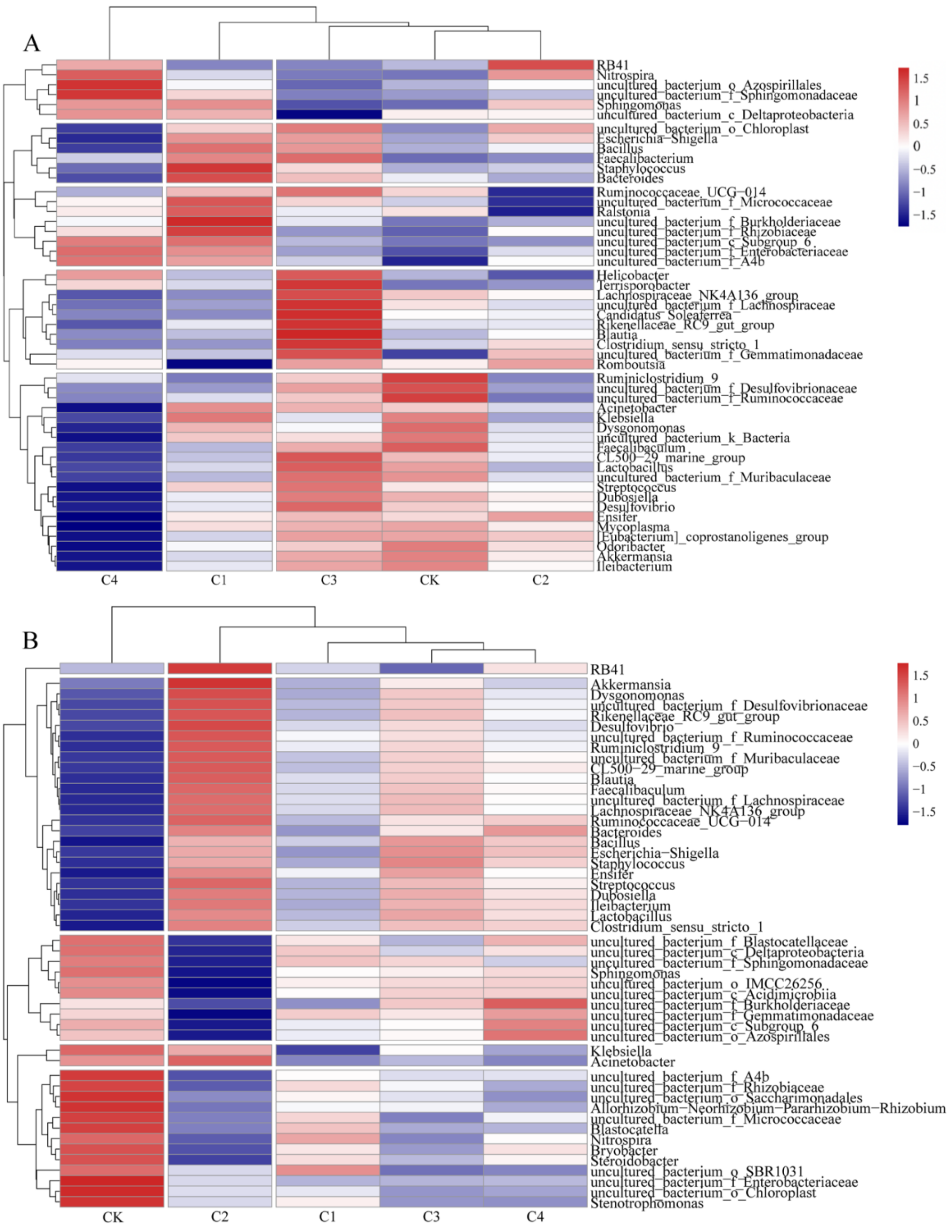

FIGURE S1 | Effect of biochar addition on the structure of soil bacterial communities. A) Changes in the bacterial communities of bulk soil at the genus level (TPO50). B) Changes in the bacterial communities of rhizosphere soil at the genus level (TPO50).

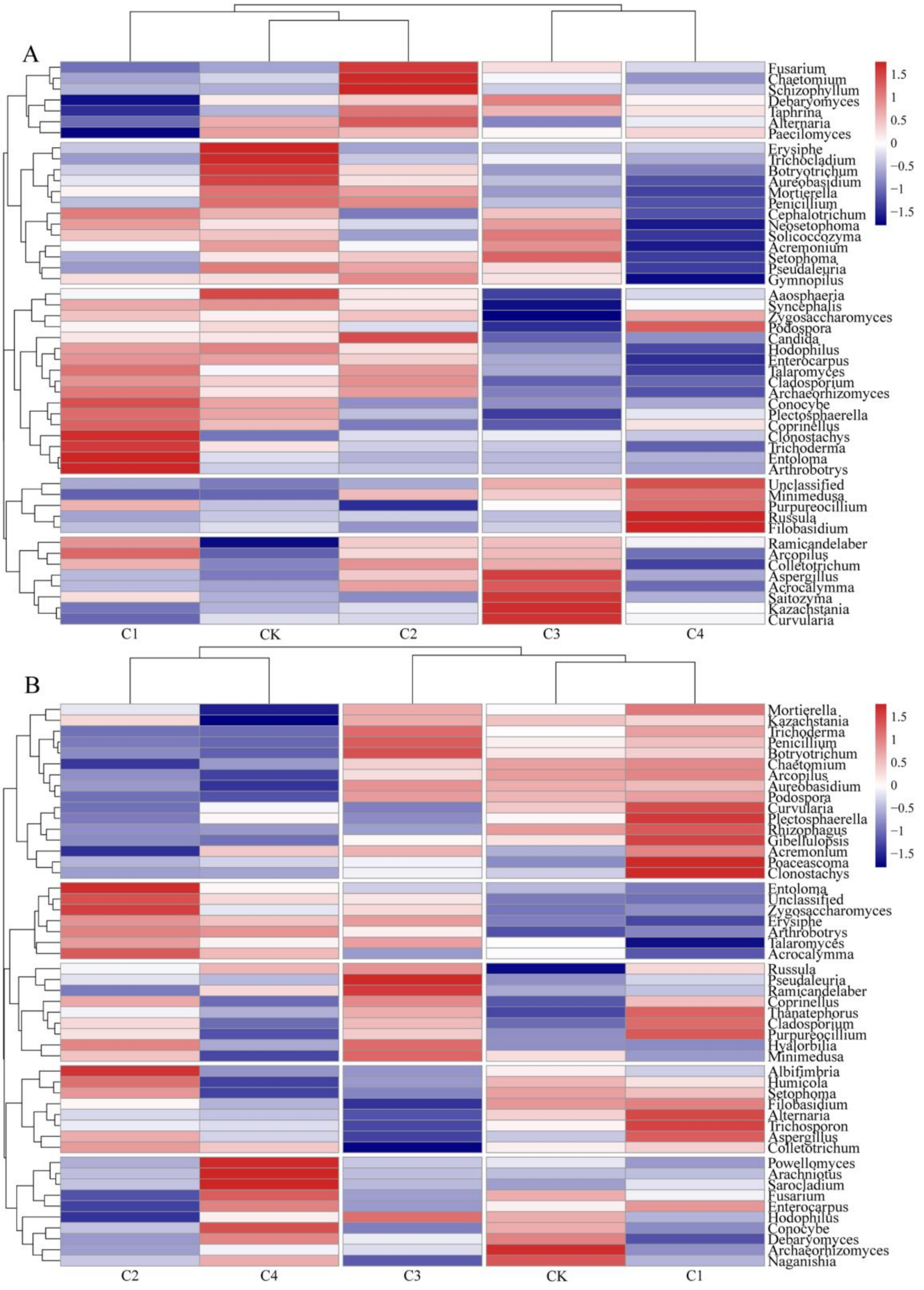

FIGURE S2 | A) Variation in the fungal communities of bulk soil at the genus level (TPO50). B) Variation in the fungal communities of rhizosphere soil at the genus level (TPO50).

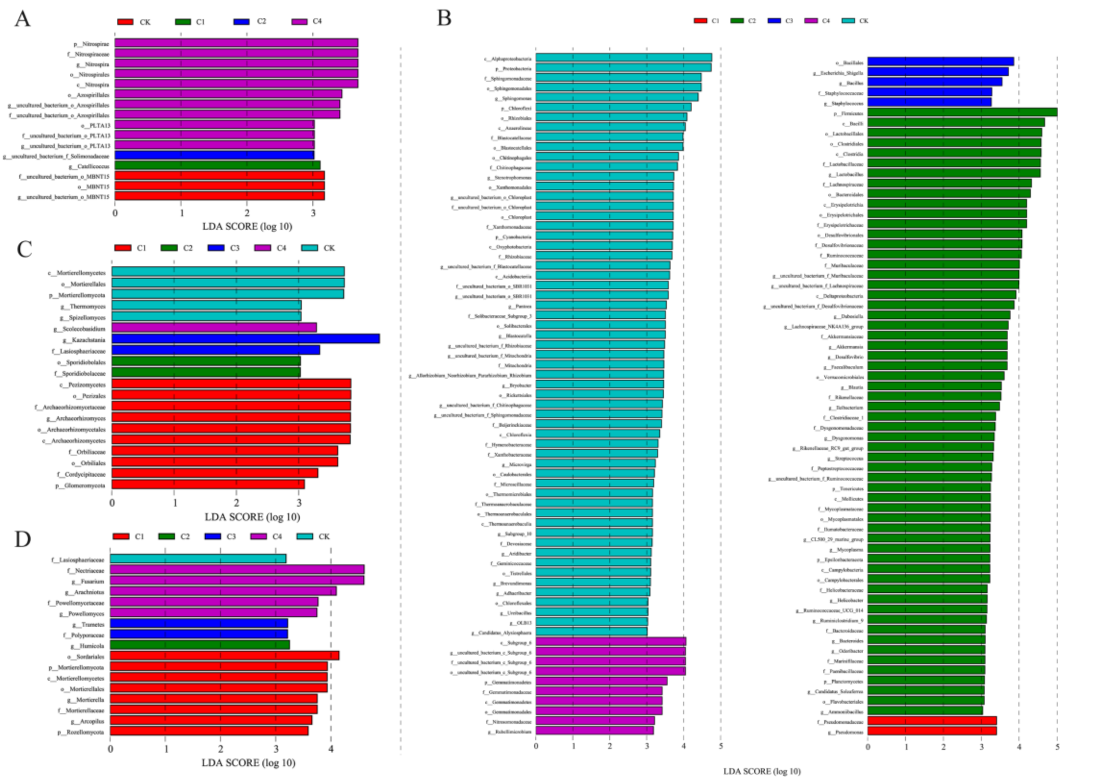

FIGURE S3 | Analysis of the effect of biochar addition on soil microbial communities at the phylum and genus level using LEfSe. A) Bacterial communities in bulk soil. B) Bacterial communities in rhizosphere soil. C) Fungal communities in bulk soil. D) Fungal communities in rhizosphere soil.

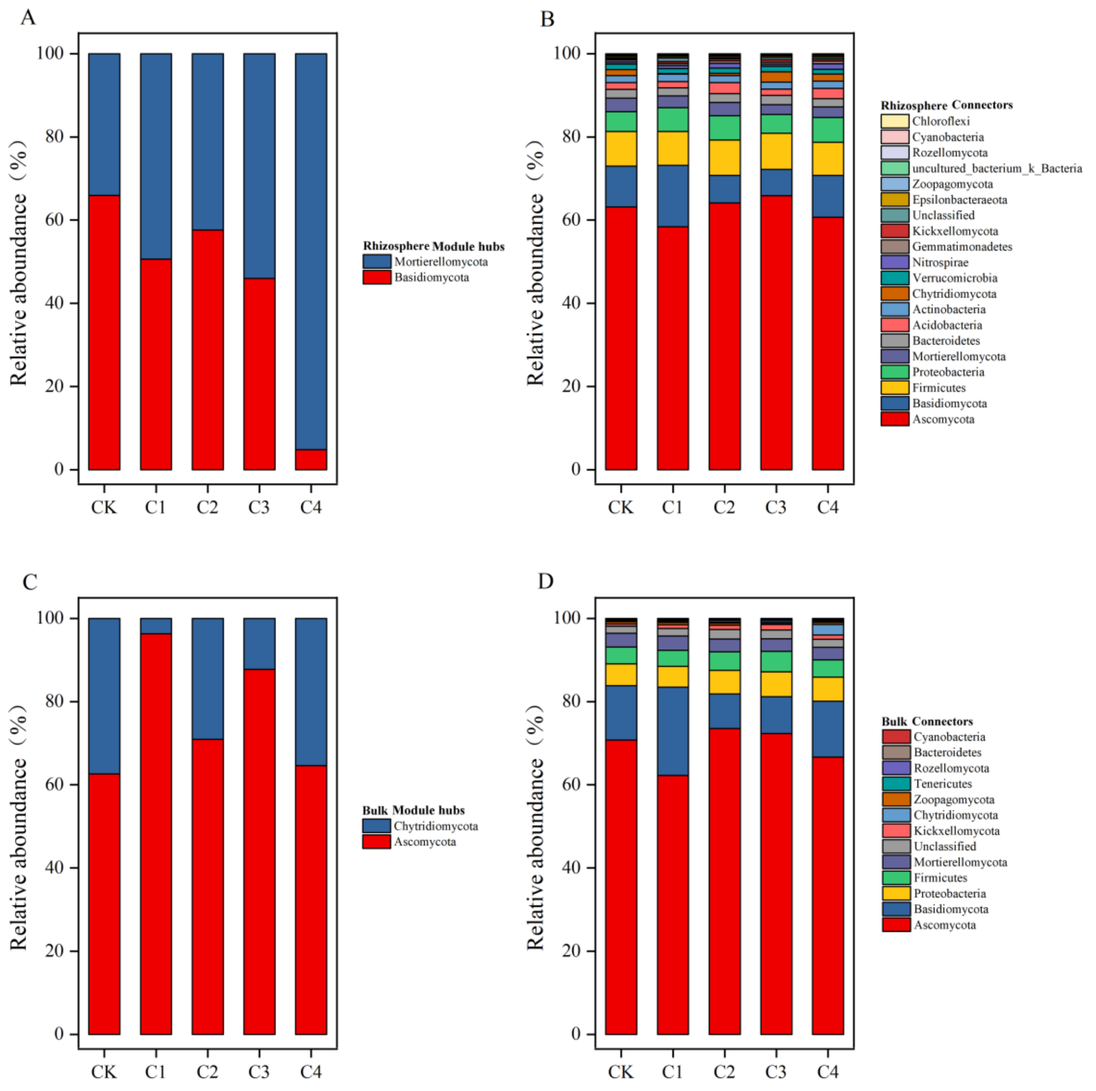

FIGURE S4 │ Composition of core microbes at the phylum level in the co-occurrence networks. A) Module hubs taxa of the rhizosphere soil co-occurrence network; B) Connectors taxa of the rhizosphere soil co-occurrence network; C) Module hubs taxa of the bulk soil co-occurrence network; and D) Connectors taxa of the bulk soil co-occurrence network.

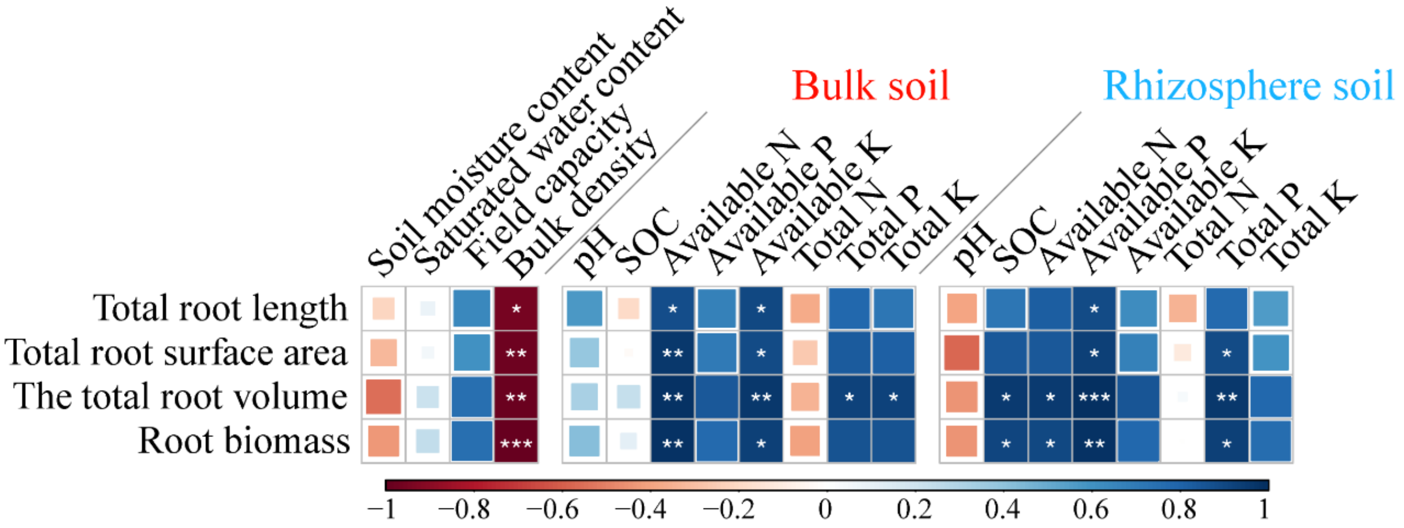

FIGURE S5 | Relationship between soil properties and maize root growth. *:P<0.05; **:P<0.01.
